# Bioinformatics-based investigation on the genetic influence between SARS-CoV-2 infections and idiopathic pulmonary fibrosis (IPF) diseases, and drug repurposing

**DOI:** 10.1038/s41598-023-31276-6

**Published:** 2023-03-22

**Authors:** Md. Ariful Islam, Md. Kaderi Kibria, Md. Bayazid Hossen, Md. Selim Reza, Samme Amena Tasmia, Khanis Farhana Tuly, Md. Parvez Mosharof, Syed Rashel Kabir, Md. Hadiul Kabir, Md. Nurul Haque Mollah

**Affiliations:** 1 Bioinformatics Lab(Dry), Department of Statistics, University of Rajshahi, Rajshahi, 6205 Bangladesh; 2 School of Business, University of Southern Queensland, Toowoomba, QLD 4350 Australia; 3 Department of Biochemistry and Molecular Biology, University of Rajshahi, Rajshahi, 6205 Bangladesh

**Keywords:** Computational biology and bioinformatics, Drug discovery

## Abstract

Some recent studies showed that severe acute respiratory syndrome coronavirus 2 (SARS-CoV-2) infections and idiopathic pulmonary fibrosis (IPF) disease might stimulate each other through the shared genes. Therefore, in this study, an attempt was made to explore common genomic biomarkers for SARS-CoV-2 infections and IPF disease highlighting their functions, pathways, regulators and associated drug molecules. At first, we identified 32 statistically significant common differentially expressed genes (cDEGs) between disease (SARS-CoV-2 and IPF) and control samples of RNA-Seq profiles by using a statistical r-package (edgeR). Then we detected 10 cDEGs (CXCR4, TNFAIP3, VCAM1, NLRP3, TNFAIP6, SELE, MX2, IRF4, UBD and CH25H) out of 32 as the common hub genes (cHubGs) by the protein–protein interaction (PPI) network analysis. The cHubGs regulatory network analysis detected few key TFs-proteins and miRNAs as the transcriptional and post-transcriptional regulators of cHubGs. The cDEGs-set enrichment analysis identified some crucial SARS-CoV-2 and IPF causing common molecular mechanisms including biological processes, molecular functions, cellular components and signaling pathways. Then, we suggested the cHubGs-guided top-ranked 10 candidate drug molecules (Tegobuvir, Nilotinib, Digoxin, Proscillaridin, Simeprevir, Sorafenib, Torin 2, Rapamycin, Vancomycin and Hesperidin) for the treatment against SARS-CoV-2 infections with IFP diseases as comorbidity. Finally, we investigated the resistance performance of our proposed drug molecules compare to the already published molecules, against the state-of-the-art alternatives publicly available top-ranked independent receptors by molecular docking analysis. Molecular docking results suggested that our proposed drug molecules would be more effective compare to the already published drug molecules. Thus, the findings of this study might be played a vital role for diagnosis and therapies of SARS-CoV-2 infections with IPF disease as comorbidity risk.

## Introduction

The severe acute respiratory syndrome corona virus 2 (SARS-CoV-2) is the main cause of COVID-19 pandemic that brings a major threat for human life as well as economy around the world^1,2^. It was first detected from Wuhan town in China at the end of 2019 and rapidly spread all over the world with several symptoms like as cough, fever, and pneumonia diarrhea, severe respiratory diseases and become a complex and deadly health concern^3,4^. The World Health Organization (WHO) announced it as a momentous pandemic of twenty-first century on March 11, 2020. It is highly homologous to the SARS coronavirus (SARS-CoV) which was responsible for the respiratory pandemic during the 2002–2003 period^5,6^. Coronaviruses are single-stranded RNA viruses of ~ 30 kb. Based on their genomic structures, they are generally classified into four genera known as α, β, γ, and δ. The life cycle of SARS-CoV-2 with the host consists of the five steps classified as attachment, penetration, biosynthesis, maturation and release. The SARS-CoV-2 enters in to the host cells through the membrane fusion or endocytosis (penetration) after binding to the host receptor proteins (attachment). Once viral proteins (biosynthesis) including the major protease (MPro/3ClPro), the papain-like protease (PLpro) and the RNA-dependent RNA polymerase (RdRp) are released inside the host cells, viral RNA enters to the nucleus for replication. Thus, create new viral particles (maturation) and released. Coronaviruses consist of four structural proteins; Spike (S), membrane (M), envelop (E) and nucleocapsid (N)^7^. The spike (S) protein of SARS-CoV-2 interacts with the host ACE2 (angiotensin-converting enzyme 2) receptor protein to stimulate the infection^8–10^. ACE2 is highly expressed in lung, heart, kidney, ileum and bladder^11,34^. In the case of lung, ACE2 is highly expressed in lung epithelial cells which leads the interstitial lung damage including epithelial and endothelial injury with excessive fibroproliferation^12^. Until September 2022, there have been around 614,825,354 confirmed cases of COVID-19, including 6,536,284 million deaths^13^. Though different vaccination programs reduced the severity of SARS-CoV-2 infections worldwide, however, it is yet one of the most severe risk factor for some chronic diseases including idiopathic pulmonary fibrosis (IPF) disease, since they stimulate each other very much^12,14^. The IPF disease is a chronic, progressive lung syndrome which leads to the respiratory collapse and decline the lung function^12^. The primary symptoms of IPF are dry cough and breathing complexity^15^. The average survival time of a patients suffering from IPF is approximately 3 years after the first diagnosis and therapies. Initially, IPF has been considered as a chronic inflammatory process^16^, but recent studies showed that abnormally activated alveolar epithelial cells (AECs) are the main factor responsible for the fibrotic response because they release cytokines that stimulate the fibroblasts^17^. IPF is typified by the progressive and fatal accumulation of fibroblasts and extracellular matrix (ECM) in the lung, leading to distortion of the lung architecture and reduction in lung function. So, Individuals with inflammatory lung disease are at a higher risk of death from COVID-19^18,19^. Therefore, identification of SARS-CoV-2 and IPF diseases causing shared genes is required for diagnosis and therapies of COVID-19 patients with IPF disease as comorbidity.

**Figure 1:**
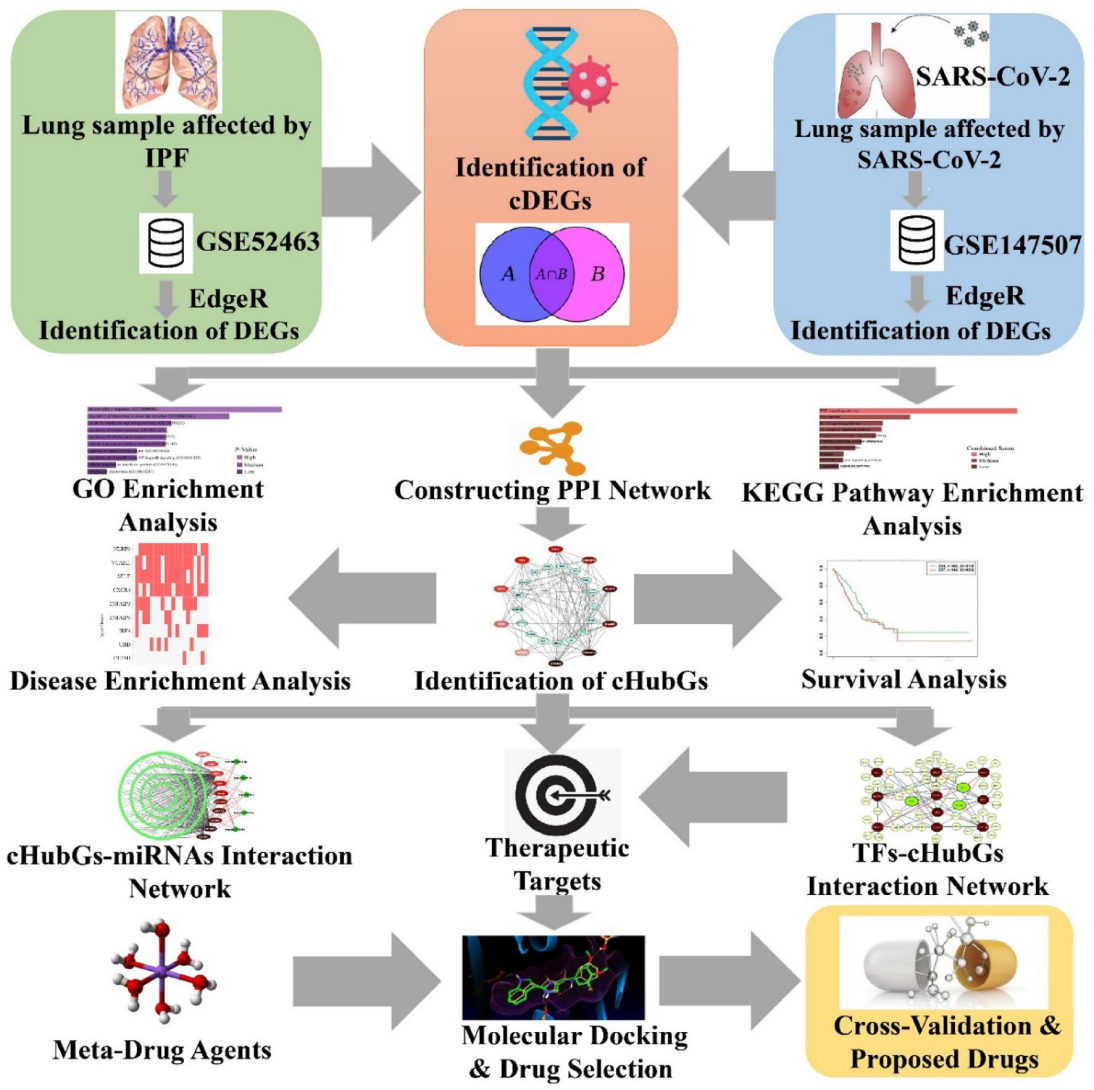
The pipeline of this study.

Taz et al.^14^ tried to identify shared genomic biomarkers for diagnosis and therapies of COVID-19 patients with IPF disease as comorbidity. They analyzed bulk RNA-Seq profiles for SARS-CoV-2 infections and microarray gene-expression profiles for IPF disease and found only 11 common differentially expressed genes (cDEGs) to separate both COVID-19 and IPF patients from control samples. They identified top-ranked 5 genes as common hub-genes (cHubGs) by protein–protein interaction (PPI) analysis, where only 2 cHubGs were detected from the cDEGs and the rest 3 cHubGs did not belongs to their cDEGs-set. Also, they did not examine their common differential expression patterns by from any other databases, which indicates that their cHubGs-set were not so representative of their cDEGs-set. Another drawback in their study was that they used microarray data instead of RNA-Seq data for identification of differentially expressed genes (DEGs) between IPF disease and control samples, though RNA-Seq data perform better than microarray data in identifying DEGs^20^. Therefore, in this study, an attempt was made to explore comparatively more representative and effective cHubGs-set for SARS-CoV-2 and IPF diseases from RNA-Seq profiles for diagnosis and therapies of COVID-19 patients with IPF disease as comorbidity by using the integrated bioinformatics analyses. The pipeline of this study is given in Fig. 1.

## Materials and methods

### Data sources and descriptions

We used both original data and meta-data to reach the goal of this study as described below.

### Collection of RNA-Seq profiles for SARS-CoV-2 infections, IPF disease and control samples

We collected RNA-Seq profiles for SARS-CoV-2 infections, IPF disease and control samples from the National Center for Biotechnology Information (NCBI) Gene Expression Omnibus (GEO) database (http://www.ncbi.nlm.nih.gov/geo/) to explore both diseases causing common genes. The SARS-CoV-2 infected patient’s RNA-Seq profiles were downloaded from GPL18573 platform of Illumina NextGen 500 (Homo sapiens) with the GEO accession numbers GSE147507 published by Blanco-Melozet al.^21^. We collected 18 case and 29 control samples of RNA-Seq profiles from 3 cell lines (1) normal human bronchial epithelium, alveolar cells, transformed lung-derived Calu-3 cells of lung tissues for SARS-CoV-2 infections. On the hand, the IPF patient’s RNA-Seq profiles were downloaded from GPL11154 platform of Illumina HiSeq 2000 (Homo sapiens) with the GEO accession numbers GSE52463 published by Nance et al.^22^. This dataset contained 16 samples including 8 IPF and 8 control samples collected from lung tissues.

### Collection of meta-drug agents for exploring candidate drugs

Beck et.al.^23^ suggested SARS-CoV-2 main protease (3CLpro)-guided top-ranked 90 drug molecules out of 3410 FDA approved anti-viral drugs for the treatment against COVID-19 by molecular docking analysis. In our study, we collected their suggested top-ranked 90 drug molecules as the meta-drug agents [see Table S1(I)]. We also collected host transcriptome-guided 95 meta-drug agents that are recommended for COVID-19 or IPF diseases [see Table S1(II)] in different published articles. Thus, we considered total 90 + 95 = 185 drugs to explore most probable candidate drug agents by molecular docking with our proposed receptors.

### Collection of independent meta-receptors for in-silico validation of the proposed drugs

We selected the top-ranked 11 hub-genes (independent meta-receptors) as the independent meta-receptors associated with COVID-19 and IPF disease by reviewing 23 published articles [see Table S1(III)] for *in-silico* validation of the proposed drug molecules by molecular docking.

### Identification of DEGs from RNA-Seq profiles by using edgeR

To explore DEGs between case and control samples from RNA-Seq profiles GSE147507 and GSE52463 of NCBI-GEO database, we considered a popular statistical approach known as edgeR. In order to introduce edgeR, let ${X}_{gi}$ denote the total number of read counts for *g*th gene (*g* = 1, 2, …, *G*) in the *i*th sample (*i* = 1, 2, …, *n)*, which is assumed to be followed negative binomial (NB) distribution in the edgeR setting^24,25^. That is, $X\sim NB\left({\mu }_{gi}, {\delta }_{g}\right),$ where the parameters are described by the mean and variance as ${\mu }_{gi}=E\left({X}_{gi}\right)={M}_{i}{\pi }_{gi}$ and $V\left({X}_{gi}\right)={\mu }_{gi}(1+{\mu }_{gi}{\delta }_{g})$, respectively. Here, ${M}_{i}$ is the total number of short reads of RNA-Seq profiles, ${\pi }_{gi}$ denote the fraction of all cDNA fragments for *g*th gene in the *i*th sample so that $\sum_{g=1}^{G}{\pi }_{gi}=1,$ ${\delta }_{g}$ is the squired coefficient of variation of ${\pi }_{gi}$ based on the replicates *i*. The NB distribution convert to Poisson distribution when ${\delta }_{g}=0$. According to the generalized linear model (GLM) approach, the mean response, ${\mu }_{gi}$, is linked to a linear predictor as ${Log(\mu }_{gi})={{{\varvec{z}}}_{i}^{T}{\varvec{\beta}}}_{g}{+Log(M}_{i}),$ where ***z***_***i***_ is the *i*th column of the design matrix (***Z***) including the covariates (e.g. batch effects, experimental conditions, etc.), ${{\varvec{\beta}}}_{g}$ is a q × 1 vector of regression parameters for differential expression patterns. The regression vector ${{\varvec{\beta}}}_{g}$ is estimated by maximum likelihood estimation (MLE) and calculated iterative way as

$${\beta }_{g}^{new}={\beta }_{g}^{old}+{({Z}^{T}{D}_{g}Z)}^{-1}{Z}^{T}{u}_{g}$$

where ${D}_{g}$ is the diagonal matrix of working weights, ${u}_{gi}=({x}_{gi}-{\mu }_{gi})/(1+{\delta }_{g}{\mu }_{gi})$. The dispersion parameter ${\delta }_{g}$ is calculated as

$${\widehat{\delta }}_{g}=\mathrm{argmax}\left\{{\mathrm{APL}}_{g}\left({\updelta }_{g}\right)+\tau .{\mathrm{APL}}_{g}^{S}\left({\updelta }_{g}\right)\right\},$$

where

$${\mathrm{APL}}_{g}\left({\updelta }_{g}\right)=L\left({\updelta }_{g};{\mathrm{X}}_{g },{\widehat{\beta }}_{g}\right)-\frac{1}{2}Log|{I}_{g}|$$

is the adjusted profile likelihood (APL) in terms of ${\delta }_{g}$, penalized for the estimation of the regression parameters ${\beta }_{g}$, ${\mathrm{X}}_{g}$ is the vector of counts for gene *g*,${\widehat{\beta }}_{g}$ is the estimated coefficient vector, *L*(·) is the log-likelihood function, ${I}_{g}$ is the Fisher information matrix and |·| is the determinant, $\tau$ is the prior degree of freedom afforded to the shared likelihood and

$${\mathrm{APL}}_{g}^{S}\left({\updelta }_{g}\right)=\frac{1}{|C|}\sum_{k\in C}{\mathrm{APL}}_{k}({\updelta }_{g}).$$

Then the edgeR approach allows us for testing the significance of any parameter or any contrast or linear combination of the parameters in the linear model. Gene-wise hypothesis testing are conducted by computing likelihood ratio (LRT) statistic $L({\theta }_{g,0}|X)/L({{\varvec{\theta}}}_{g}|X)$ to compare the null hypothesis (H_0_) that ${{\varvec{\beta}}}_{{\varvec{g}}}=0$ (insignificant gene) against the two-sided alternative (H_1_), ${\varvec{\beta}}\ne 0$ (indicating DEGs), where ${{\varvec{\theta}}}_{g}=\{{\varvec{\beta}},\boldsymbol{ }\delta \}$ and ${\theta }_{g,0}=\delta$. The log-LRT follows asymptotically chi-square distribution under H_0_. Adjusted *P*.values are computed to control the FDR (false discovery rate) by the Benjamini and Hochberg approach^26^. We implemented this algorithm to identify DEGs from our downloaded RNA-Seq count datasets GSE147507 and GSE52463 for SARS-CoV-2 and IPF diseases, respectively, by using edgeR, an R-package in Bioconductor^25^. To separate up and down-regulated DEGs, we for the combined data as follows

$${\text{DEG}}_{g} = \left\{ {\begin{array}{*{20}l} {{\text{Upregulated}},} & {{\text{if}}\,{\text{adj}}.P_{g} .{\text{value}} < 0.05\,{\text{and}}\,{\text{Log}}_{2} ({\text{aFC}})_{g} > + 1.0} \\ {{\text{Downregulated}},} & {{\text{if}}\;{\text{adj}}.P_{g} .{\text{value}} < 0.05\,{\text{and}}\,{\text{Log}}_{2} ({\text{aFC}})_{g} < - 1.0} \\ \end{array} } \right.{\text{ }}$$

where $\mathrm{adj}.{P}_{g}.\mathrm{value}$ is the adjusted *P*.value and $({\mathrm{aFC})}_{\mathrm{g}}={\overline{X} }_{g}^{\mathrm{case}}/{\overline{X} }_{g}^{\mathrm{control}}$: fold change on average expressions, for *g*th gene.

## Identification of SARS-CoV-2 and IPF diseases causing common DEGs (cDEGs)

Let ${\mathrm{C}}_{\mathrm{UR}}$ and ${\mathrm{C}}_{\mathrm{DR}}$ indicates the upregulated and downregulated DEGs-sets respectively for Covid-19 patients. Again let ${\mathrm{I}}_{\mathrm{UR}}$ and ${\mathrm{I}}_{\mathrm{DR}}$ be the upregulated and downregulated DEGs-sets respectively, for IPF patients. Then, we defined common upregulated gene-set as ${G}_{UR}=({C}_{UR}\cap {I}_{UR})$ and common downregulated as ${G}_{DR}=({C}_{DR}\cap {I}_{DR})$. Finally, we considered common DEGs (cDEGs) set as $\mathrm{cDEG}=({\mathrm{G}}_{\mathrm{UR}}\cup {\mathrm{G}}_{\mathrm{DR}})$ that can differentiate both group of patients from the control samples. Therefore, in this study, we considered this cDEGs-set for further investigation.

### Protein–protein interaction (PPI) network analysis of cDEGs

In order to explore both SARS-CoV-2 and IPF diseases causing common hub-genes (cHubGs), we performed protein–protein interaction (PPI) network analysis of cDEGs by using the STRING databases^27^. To build the PPI network, the distance "D" between the proteins (u, v) is computed as,

$$D\left(u,v\right)= \frac{2 |{N}_{u}\cap {N}_{v}|}{\left|{N}_{u}\right|+|{N}_{v}|}$$

where ${N}_{u}$ and ${N}_{v}$ are the neighbor sets of u and v, respectively. To improve the quality of PPI networks, we used the Cytoscape web-tool^28^. The Cytoscape plugin cytoHubba was used to select the Hub-Genes (HubGs) or Hub-Proteins (HubPs) from PPI networks^28,29^. The PPI network provides a number of nodes and edges, which indicates proteins and their interactions, respectively. The HubGs were selected from the PPI network by using five topological measures including Degree (Deg)^30^, BottleNeck (BN)^31^, Betweenness (BC)^32^, Stress (Str)^33^, and Closeness (Clo)^34^.

### Regulatory network analysis of cHubGs

To explore key transcription factors (TFs) and micro-RNAs (miRNAs) as the transcriptional and post-transcriptional regulators of cHubGs, we performed TFs-cHubGs, miRNAs-cHubGs, and TFs-miRNAs-cHubGs interaction networks based on the JASPAR^35^, TarBase^36^ and RegNetwork^37^ databases, respectively by using the NetworkAnalyst web-tool^38^. The Cytoscape software^28^ was used to improve the quality of networks. The key regulators were selected by using two topological measures (Degree (Deg)^30^ and Betweenness (BC)^32^) of networks.

### GO terms and KEGG pathway enrichment analysis of cDEGs highlighting cHubGs

To explore the pathogenetic processes of cDEGs, we performed enrichment analysis of cHubGs with different gene ontology (GO) terms (BPs: biological processes, MFs: molecular functions and CCs: cellular components)^39^ and Kyoto encyclopedia of genes and genomes (KEGG) pathways^40^. Here, BPs are different types of molecular activities that are essential for the functioning of integrated living entities including cells, tissues, and organs. MFs are different types of fundamental molecular activity of a gene product, including catalysis. CCs are different types of components of a cell or the extracellular space. The KEGG pathway database consists of a set of pathway maps that represent the molecular interaction and relationship networks for genetic information processing, metabolism, human diseases, and drug development. Let *Si* denote the reference/annotated gene-set in the *i*th GO-term or KEGG-pathway, *Mi* denote the total number genes in *S*_*i*_ (*i* = 1, 2,…, *r*); *N* denote the total number of reference/annotated genes that construct the whole-set $S=\bigcup_{i=1}^{r}{S}_{i}={S}_{i}\bigcup {S}_{i}^{c}$ such that $N\le \sum_{i=1}^{r}{M}_{i} ;$ where ${S}_{i}^{c}$ is the complement set of *S*_*i*_. Again, let *n* represent the total number of cDEGs and *ki* represent the number of cDEGs that are a part of the annotated gene-set *S*_*i.*_ To examine the enrichment of *i*th GO-term or KEGG-pathway by the cDEGs-set, the following contingency table (Table 1) is constructed.

**Table 1:** $2\times 2$ Contingency table.

Annotated gene-set (Reference)	cDEGs-set	Not cDEGs-set	Marginal total
*S*_*i*_	*k*_*i*_	*M*_*i*_ − *k*_*i*_	*M*_*i*_
${S}_{{\varvec{i}}}^{c}$	*n* − *k*_*i*_	*N* − *M*_*i*_ − *n* + *k*_*i*_	*N* − *M*_*i*_
Marginal total	*n*	*N* − *n*	*N* (Grand total)

We considered three web tools (Enrichr^41^, DAVID^42,43^, and GeneCodis^44^) to explore significantly enriched GO-terms and KEGG pathways by using the chi-square (${\chi }^{2})$ or Fisher’s exact testing procedures. The ${\chi }^{2}$-testing procedure based on $2\times 2$ contingency table, is used in GeneCodis to calculate the *p-*values, while the Fisher’s exact testing procedure is used in both Enrichr and DAVID web-tools. Fisher’s exact testing procedure is formulated based on hypergeometric distribution. This distribution is used to estimate the probability of overlapping exactly *k*_*i*_ cDEGs with the reference genes in the *i*th GO-term or pathway (*S*_*i*_) out of *n* cDEGs. Then the *p* value for testing the significance of *k*_*i*_ cDEGs in *i*th GO-term or pathway is calculated as,

$${p}_{i}= 1 - \sum\limits _{{j = 0}}^{{k_{i} }}\frac{\left(\genfrac{}{}{0pt}{}{Mi}{j}\right)\left(\genfrac{}{}{0pt}{}{N-Mi}{n-j}\right)}{\left(\genfrac{}{}{0pt}{}{N}{n}\right)}, i=\mathrm{1,2},\dots ,r.$$

The *k*_*i*_ cDEGs in *S*_*i*_ is considered as a significantly enriched gene-set if its adjusted *p* value (*p*_*i*_) < 0.05 at 5% level of significance. We adjusted the *p* values for each of three procedures by using the Benjamini and Hochberg procedure^26^.

### Association of cHubGs with other disease risk

We performed diseases-cHubGs association analysis by using the Enrichr web tool^45^ with DisGeNET database^46,47^ to explore other diseases that can increase the severity of COVID-19 and IPF both diseases through cHubGs. The DisGeNET database is a comprehensive discovery platform to address association of a gene-set with different disease risk^47^. The necessary data for this database has been collected from different online sources including UniProt, Comparative Toxicogenomics Database (CTD), Mouse Genome Database (MGD), Rat Genome Database (RGD) and, peer-reviewed publications on Genome Wide Association Studies (GWAS), Genetics Association Database (GAD), literature of human gene-disease networks (LHGDN), and the BeFree datasets. To measure the gene-disease association (GDA), the DisGeNET web-tool compute a score (S) by using the following formula.

$$S= {(S}_{\mathrm{UNIPORT}}+{S}_{\mathrm{CTD \,human}})+{(S}_{\mathrm{Mouse}}+{S}_{\mathrm{Rat}})+{(S}_{\mathrm{GDA}}+{\mathrm{S}}_{\mathrm{LHGDNA}}+{S}_{\mathrm{BeFree}})$$

where

$$S_{{{\text{UNIPORT}}}} = \left\{ {\begin{array}{*{20}l} {0.3,} & {{\text{if}}\,{\text{the}}\,{\text{association}}\,{\text{has}}\,{\text{been}}\,{\text{decleared}}\,{\text{in}}\,{\text{Unipropt}}} \\ {0,} & {{\text{otherwise}}} \\ \end{array} } \right.$$

$$S_{{{\text{CTD\,human}}}} = \left\{ {\begin{array}{*{20}l} {0.3} & {{\text{if}}\,{\text{the}}\,{\text{association}}\,{\text{has}}\,{\text{been}}\,{\text{decleared}}\,{\text{in}}\,{\text{CTD}}\;{\text{human}}} \\ {0,} & {{\text{otherwise}}} \\ \end{array} } \right.$$

$$S_{{{\text{Rat}}}} = \left\{ {\begin{array}{*{20}l} {0.3,} & {{\text{if}}\,{\text{the}}\,{\text{association}}\,{\text{has}}\,{\text{been}}\,{\text{decleared}}\,{\text{in}}\,{\text{CTD}}\;{\text{or}}\,{\text{RGD}}\,{\text{rat}}\;{\text{dataset}}} \\ {0,} & {{\text{otherwise}}} \\ \end{array} } \right.$$

$$S_{{{\text{Mouse}}}} = \left\{ {\begin{array}{*{20}l} {0.3,} & {{\text{if}}\,{\text{the}}\,{\text{association}}\,{\text{has}}\,{\text{been}}\,{\text{decleared}}\,{\text{in}}\,{\text{CTD}}\,{\text{or}}\,{\text{RGD}}\,{\text{mouse}}\,{\text{dataset}}} \\ {0,} & {{\text{otherwise}}} \\ \end{array} } \right.$$

$$S_{L} = \left\{ {\begin{array}{*{20}l} {0.1,} & {if\,\frac{{n_{{gd}} \times 100}}{{N_{L} }} \ge \,{\text{max}}} \\ {\frac{{n_{{gd}} \times 100}}{{N_{L} }},} & {if\,\frac{{n_{{gd}} \times 100}}{{N_{L} }} < max} \\ \end{array} } \right.$$

and *L*: LHGDN, GAD, or BeFree, ${n}_{gd}$ is the number of publications reported a GDA in the source and ${N}_{L}$ is the total number of publications in the source.

$${\text{max}} = \left\{ {\begin{array}{*{20}l} {0.080} & {if\;L = GAD} \\ {0.06} & {if\;L = {\text{LHGDN}}\;{\text{or}}\;{\text{BeFree}}} \\ \end{array} } \right.$$

Obviously, the DisGeNET score (S) lies between 0 to 1.

## Prognostic performance of cHubGs

To investigate the prognostic performance of cHubGs with lung cancer, we performed multivariate survival analysis of lung cancer patients based on the expressions of cHubGs by using SurvExpress web*-*tool^48^. The TCGA database (https://tcga-data.nci.nih.gov) is used in SurvExpress web*-*tool for survival analysis with a gene-set. In this analysis, patient samples were divided in to low-risk and high-risk groups by using the median of risk-scores (RSs) which is defined by the linear component of the Cox regression model. That is, ${\mathrm{RS}=\upbeta }_{1}{\mathrm{X}}_{1}+{\upbeta }_{2}{\mathrm{X}}_{2}+\dots +{\upbeta }_{\mathrm{n}}{\mathrm{X}}_{\mathrm{n}}$, where ${\mathrm{X}}_{\mathrm{i}}$ is the expression of *i*th gene, ${\upbeta }_{\mathrm{i}}$ is calculated by using the Cox regression approach^49^. Generally, a patient whose risk-score (RS) is greater than median of RSs was considered in the high-risk group, otherwise, the patient belongs to the low-risk group. Then the Kaplan–Meier survival plot for each risk group is constructed for each risk group. The significant difference between two risk groups was investigated by using the concordance indexes (CI), hazard-ratio (HR) and log-rank test^50^. The significance level was set to *p* value < 0.05.

### Drug repurposing by molecular docking

To explore cHubGs-guided FDA approved repurposable drug molecules for the treatment against SARS-CoV-2 infections in presence of IPF risk, we employed molecular docking analysis of cHubGs and its TFs with 185 drug agents as introduced previously in the data sources and descriptions section [see Table S1(I, II)]. The molecular docking analysis requires 3-Dimensional (3D) structures of both receptor proteins and drug agents/ligands. We downloaded 3D structure of all targeted proteins from Protein Data Bank (PDB)^51^, AlphaFold Protein Structure Database^52^ and SWISS-MODEL^53^. The 3D structures of all drug agents were downloaded from PubChem database^54^. The 3D structures of the target proteins were visualized by using the Discovery Studio Visualizer 2019^55^. Further, the receptor protein was prepared by removing ligand heteroatoms and water molecules and by addition of polar hydrogens on AutoDock tools 1.5.7^56^. The drug agents/ligands were prepared by setting the torsion tree and rotatable, nonrotatable bonds present in the ligand through AutoDock tools 1.5.7. Then, pairwise binding affinities between the target proteins and drug agents were calculated using the AutoDock Vina^57^. The exhaustiveness parameter was set to 10. Discovery Studio Visualizer 2019^55^ and PyMol^58^ were used to analyze the docked complexes for its surfaces, types and distances of non-covalent bonds. Let *B*_*ij*_ is the binding affinity (BA) score corresponding to the *i*th receptor (*i* = 1, 2, … , *p*) and *j*th drug (*j* = 1, 2, … , *q*). The receptors and drug agents were ordered by the decreasing order of their average BA scores for selecting the top-ordered potential drug agents. We compared the binding performance of our suggested drugs with previously suggested candidate drugs by Taz et al.^14^ by molecular docking analysis against the Taz et al., suggested receptors as well as top-ranked independent receptors. Finally, we looked into the effectiveness of our suggested drugs with randomly selected independent receptor proteins that were not associated with SARS-CoV-2 infections in the presence of IPF risk.

## Results

### Identification of cDEGs

**Figure 2:**
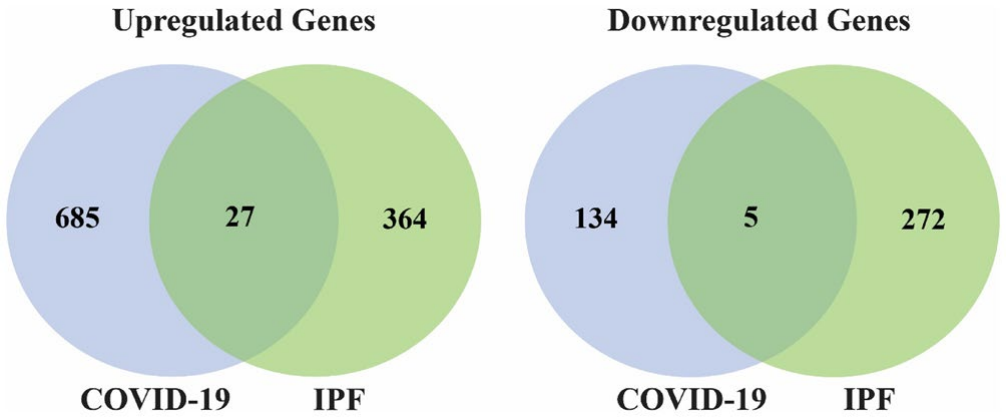
Venn diagrams for visualizing the upregulated and downregulated cDEGs that can separate both COVID-19 and IPF patients from the control samples.

The dataset GSE147507 was analyzed by using the edgeR r-package to identify DEGs between SARS-CoV-2 infections and control samples. A total of 851 DEGs were identified by satisfying the cutoff criteria of adjusted *p* value < 0.05 and |log_2_(aFC)|> 1, where 712 DEGs are upregulated and 139 DEGs are downregulated. The GSE52463 dataset was analyzed to identify DEGs between IPF diseases and control samples. A total of 668 DEGs were found according to the same criteria, where 391 DEGs are upregulated and 277 are downregulated. Then we commonly found 27 upregulated cDEGs {*TNFAIP3, SELE, MX2, PTX3, CH25H, UBASH3A, EREG, BIRC3, ZBP1, NLRP3, LIF, PRDM1, ADM, VCAM1, FOSL1, CXCR4, CCL22, IRF4, SLC5A5, SYTL3, ADAMTS4, UBD, CCL17, CPNE5, TNFAIP6, IKZF3, TNIP3*} and 5 downregulated cDEGs {*MVD, KIF12, RAAG1GAP, SLC27A3, TMEM160}* that can separate both COVID-19 and IPF patients from the control samples (see Fig. 2).

### PPI network analysis of cDEGs for identification of cHubGs

**Figure 3:**
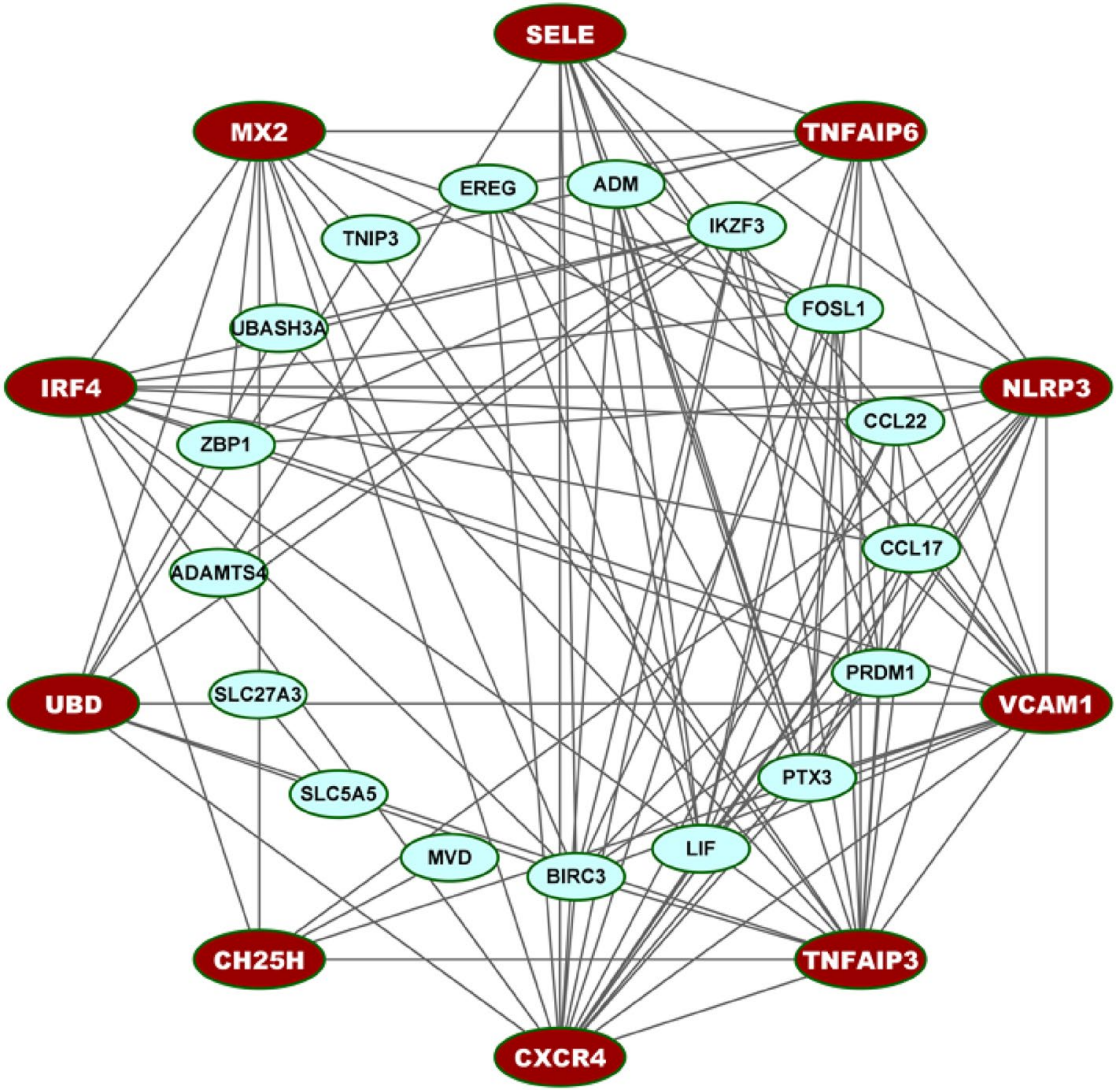
Protein–protein interaction (PPI) network of cDEGs for COVID-19 and IPF disease to identify cHubGs. The red color nodes indicate the cHubGs.

The PPI network of cDEGs was constructed using STRING database (Fig. 3) which contains 27 nodes and 115 edges. We selected top ranked 10 cHubGs {CXCR4, TNFAIP3, VCAM1, NLRP3, TNFAIP6, SELE, MX2, IRF4, UBD, CH25H} by applying five topological measures (Deg, BN, BC, Str and Clo) in the PPI network (see Table S2).

### The regulatory network analysis of cHubGs

**Figure 4:**
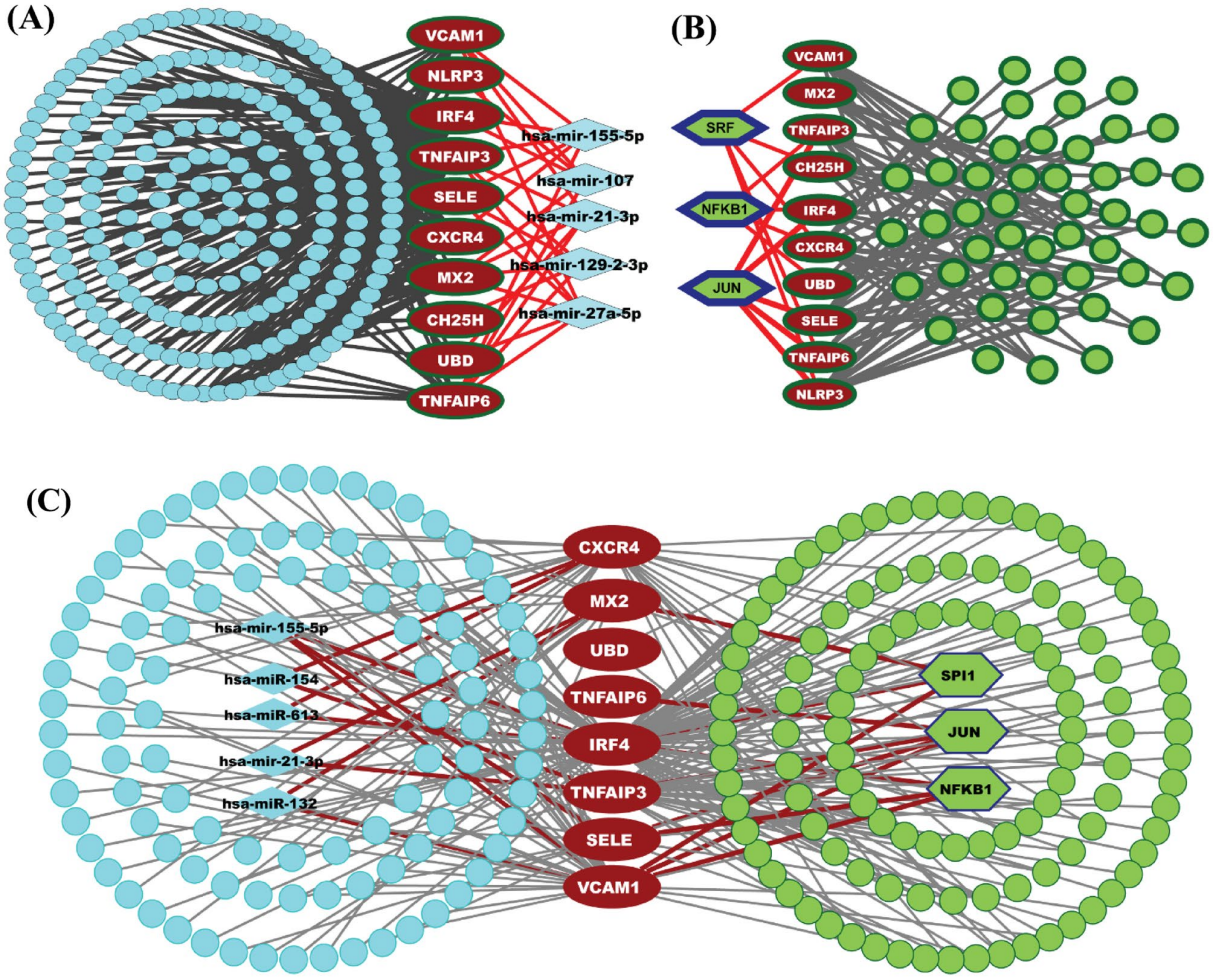
The regulatory network of cHubGs with TFs and miRNAs. The red, light blue and green color nodes represent the cHubGs, TFs and miRNAs. The names of cHubGs, key regulatory TFs and miRNAs were displayed only. (**A**) The cHubGs-TFs interaction network based on JASPAR database. (**B**) The miRNA-cHubGs interaction network based on TarBase database. (**C**) The cHubGs-TFs-miRNAs interaction network based on RegNetwork database.

The cHubGs-TFs interaction network detected top-ranked two sets of TFs proteins {JUN, SRF and NFKB1} and {JUN, SPI1 and NFKB1} based on JASPAR and RegNetwork databases respectively, as the key transcriptional regulatory factors for cHubGs, where two TFs proteins JUN and NFKB1were common in both-sets (see Fig. 4B,C). Based on two databases, we observed that JUN is a regulator of 7 cHubGs (TNFAIP3, TNFAIP6, IRF4, SELE, TNFAIP6, NLRP3, VAM1) and NFKB1 for 6 cHubGs (TNFAIP3, IRF4, CXCR4,UBD, SELE, VAM1). The cHubGs-miRNAs interaction network detected top-ranked two sets of miRNAs {hsa-mir-155-5p, hsa-mir-27a-5p, hsa-mir-107, hsa-mir-21-3p, hsa-mir-129-2-3p} and { hsa-mir-155-5p, hsa-mir-154, hsa-mir-613, hsa-mir-21-3p, hsa-mir-132} based on TarBase and RegNetwork databases respectively, as the key post-transcriptional regulatory factors for cHubGs, where two miRNAs hsa-mir-155-5p and hsa-mir-21-3p were common in both-sets (see Figs. 4A,C). Based on two databases, we observed that hsa-mir-155-5p regulates 6 cHubGs {IRF4, VAM1, TNFAIP3, TNFAIP6, SELE, CXCR4} and hsa-mir-21-3p regulates 5 cHubGs {VAM1, TNFAIP3, TNFAIP6, MX2, UBD}.

### GO functions and KEGG pathway enrichment analysis of cDEGs highlighting cHubGs

The Table 2 displayed the top five commonly enriched GO terms (BPs, MFs and CCs) and KEGG pathways by cDEGs with three web-tools and databases Enrichr^41^, DAVID^42,43^ and GeneCodis^44^ (*p* value < 0.001). Among the top five GO terms of BPs, four BPs including *inflammatory response, regulation of inflammatory response, response to tumor necrosis factor* and *response to cytokine* were enriched by the cHubGs-sets with all of three databases. The other BP-term (*response to virus)* was enriched by the cHubGs-sets with each of two databases (DAVID^42,43^ and GeneCodis^44^). Three MFs (*sequence-specific DNA binding, CCR chemokine receptor binding* and *chemokine activity)* out of four, were commonly enriched by the cHubGs-sets with all of three databases. The other MF-term (*ubiquitin binding*^59^*)* was enriched by the cHubGs-sets with each of two databases (Enrichr^41^ and GeneCodis^44^). Among the top five significantly enriched CCs terms, four terms including *early endosome,* extracellular space, cytosol *and* extracellular region were commonly enriched by the cHubGs-sets with two databases out of 3. Only one CC-term (*tertiary granule lumen)* was enriched by the cHubGs-sets with all of 3 databases. Interestingly, all of the top five KEGG pathways (*TNF signaling pathway, IL-17 signaling pathway, NF-kappa B signaling pathway, NOD-like receptor signaling* and *Cytokine-cytokine receptor interaction)* were commonly enriched by the cHubGs-sets with all of 3 databases.

**Table 2:** The top five commonly enriched GO terms (BPs, MFs and CCs) and KEGG pathways by cDEGs with three web-tools and databases Enrichr^41^, DAVID^42,43^ and GeneCodis^44^ (*p* value < 0.001). The cHubGs were highlighted by bold gene names. Some references were given to support the association of our detected GO-terms and pathways with the mechanisms of SARS-CoV-2 infections and IPF diseases.

	GO ID	GO term	Associated cDEGs in three databases highlighting cHubGs		
			Enrichr	DAVID	GeneCodis
Biological process	GO:0006954	*Inflammatory response*^60–65^	*CCL22; ****TNFAIP6****; ****CXCR4****; ****NLRP3****; ADM; PTX3; CCL17; ****SELE***	*CCL22, ****TNFAIP6****, TNIP3, ****NLRP3****, ****TNFAIP3****, ****CXCR4****, ADM, PTX3, ****SELE****, CCL17*	*PTX3, ****NLRP3****, ****TNFAIP6****, ****SELE****, ADM, ****CXCR4****, TNIP3, ****TNFAIP3****, CCL17, CCL22*
	GO:0009615	Response to virus^66,67^	–	***MX2****, ****CXCR4****, FOSL1, CCL22*	*FOSL1, CCL22, ****MX2****, ****CXCR4***
	GO:0050727	*Regulation of inflammatory response*^68,69^	*ZBP1; ****TNFAIP6****; ****TNFAIP3****; ****NLRP3****; ****SELE****; BIRC3*	*ZBP1, ****NLRP3****, ****SELE****, BIRC3*	*ZBP1, ****SELE****, BIRC3*
	GO:0034612	*Response to tumor necrosis factor*^70–72^	*CCL22; ****UBD****; CCL17; ****SELE***	***UBD****, ****SELE***	***UBD****, ****SELE***
	GO:0034097	Response to cytokine^73,74^	***UBD****; ****MX2****; ****CXCR4****; ****SELE***	*FOSL1, ****SELE***	***SELE***
Molecular functions	GO:0043130	*Ubiquitin binding*^59^	***TNFAIP3****; ****CXCR4***	*–*	***CXCR4****, ****TNFAIP3***
	GO:0043565	Sequence-specific DNA binding^75^	*FOSL1; ****IRF4****; ****NLRP3****; PRDM1; IKZF3*	***IRF4****, ****NLRP3****, IKZF3*	***IRF4****, ****NLRP3****, PRDM1, IKZF3, FOSL1*
	GO:0048020	CCR chemokine receptor binding^76^	*CCL22; CCL17*	*CCL22, CCL17*	*CCL22; CCL17*
	GO:0008009	Chemokine activity	*CCL22; CCL17*	*CCL22; CCL17*	*CCL22; CCL17*
Cellular component	GO:1904724	*Tertiary granule lumen*^77,78^	***TNFAIP6****; PTX3*	***TNFAIP6****, PTX3*	***TNFAIP6****;PTX3*
	GO:0005769	*Early endosome*^79^	***VCAM1****; ****CXCR4***	–	***CXCR4****, ****VCAM1***
	GO:0005615	Extracellular space^66^	*–*	*ADAMTS4, ****VCAM1****, CCL22, ****TNFAIP6****, LIF, ADM, PTX3, ****SELE****, CCL17, EREG*	*PTX3, LIF, EREG, ****TNFAIP6****, ****SELE****, ADM, ADAMTS4, ****VCAM1****, CCL17, CCL22*
	GO:0005829	Cytosol^80^	–	*ZBP1, ****MX2****, LIF, ****TNFAIP3****, IKZF3, FOSL1, ****CH25H****, ****IRF4****, ****UBD****, TNIP3, ****NLRP3****, MVD, UBASH3A, BIRC3*	***MX2****, ZBP1, ****UBD****, LIF, ****IRF4****, ****NLRP3****, ****CH25H****, UBASH3A, IKZF3, TNIP3, ****TNFAIP3****, FOSL1, BIRC3, MVD*
	GO:0005576	Extracellular region^81–83^	–	*ADAMTS4, CCL22, ****TNFAIP6****, LIF, ****NLRP3****, ADM, PTX3, CCL17, EREG*	*PTX3, LIF, ****NLRP3****, EREG, ****TNFAIP6****, ADM, ADAMTS4, CCL17, CCL22*
Pathways ID		Pathways			
KEGG	hsa04668	*TNF signaling pathway*^69,84^	***VCAM1****; LIF; ****TNFAIP3****; ****SELE****; BIRC3*	***VCAM1****, LIF, ****TNFAIP3****, ****SELE****, BIRC3*	*LIF, ****SELE****, ****TNFAIP3****, BIRC3, ****VCAM1***
	hsa04657	*IL-17 signaling pathway*^69,85–87^	*FOSL1; ****TNFAIP3****; CCL17*	***TNFAIP3****, FOSL1, CCL17*	***TNFAIP3****, FOSL1, CCL17*
	hsa04064	*NF-kappa B signaling pathway*^69,88^	***VCAM1****; ****TNFAIP3****; BIRC3*	***VCAM1****, ****TNFAIP3****, BIRC3*	***TNFAIP3****, BIRC3, ****VCAM1***
	hsa04621	*NOD-like receptor signaling*^89,90^	***TNFAIP3****; ****NLRP3****; BIRC3*	***NLRP3****, ****TNFAIP3****, BIRC3*	***TNFAIP3****;****NLRP3****;BIRC3*
	hsa04060	*Cytokine-cytokine receptor interaction*^69,91^	*CCL22; LIF; ****CXCR4****; CCL17*	*CCL22, LIF, ****CXCR4****, CCL17*	*LIF, ****CXCR4****, CCL17, CCL22*

### Association of cHubGs with other disease risks

**Figure 5:**
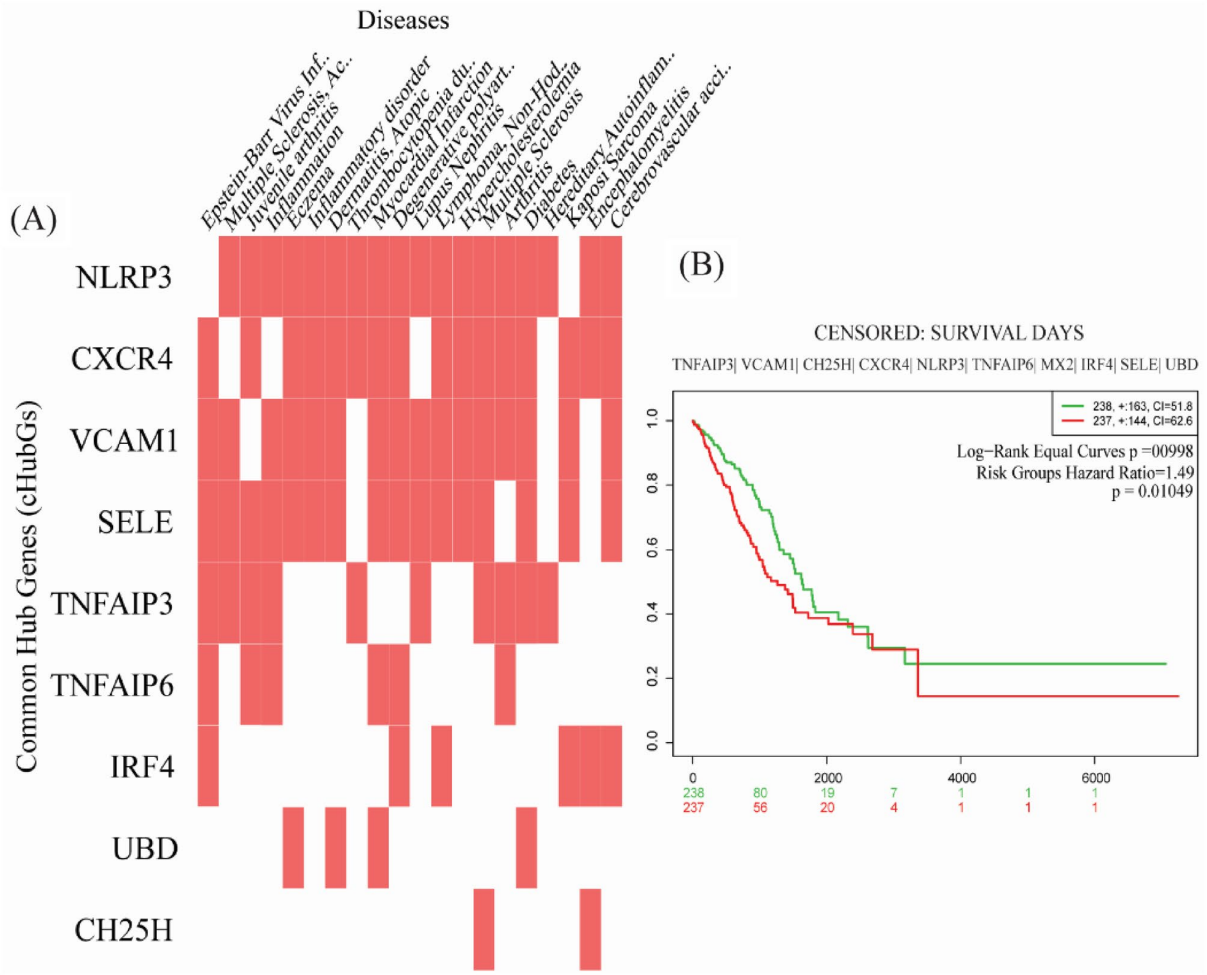
(**A**) cHubGs-disease association results. Red box indicates significant association. (**B**) The multivariate survival probability curves of lung cancer patients based on cHubGs. Red indicates the survival curve with high risk (cancer) group and green indicates the low risk (control) group.

We performed cHubGs-disease association analysis by using the Enrichr web-tool with DisGeNET database to explore other diseases that may increase the severity of both COVID-19 and IPF diseases simultaneously or separately, through the influence of cHubGs. This analysis significantly detected top-ranked 20 diseases (Epstein-Barr virus infections, Myocardial Infarction, Degenerative polyarthritis, Arthritis, Diabetes, Juvenile arthritis, Inflammation, Eczema, Dermatitis, Atopic, Lymphoma, Non-Hodgkin and Arthritis, multiple sclerosis, Acute Fulminating, Inflammatory disorder, Lupus Nephritis, Hypercholesterolemia, thrombocytopenia due to platelet alloimmunization and Hereditary Autoinflammatory ) that may stimulate both SARS-CoV-2 and IPF diseases through the influence of cHubGs (see Fig. 5A and Table S3). The multivariate survival probability curves with cHubGs significantly separated the lung cancer patients (high risk group) from the control groups (low risk group) (see Fig. 5B), which indicates the cHubGs stimulate the lung cancer also.

### Drug repurposing by molecular docking

**Figure 6:**
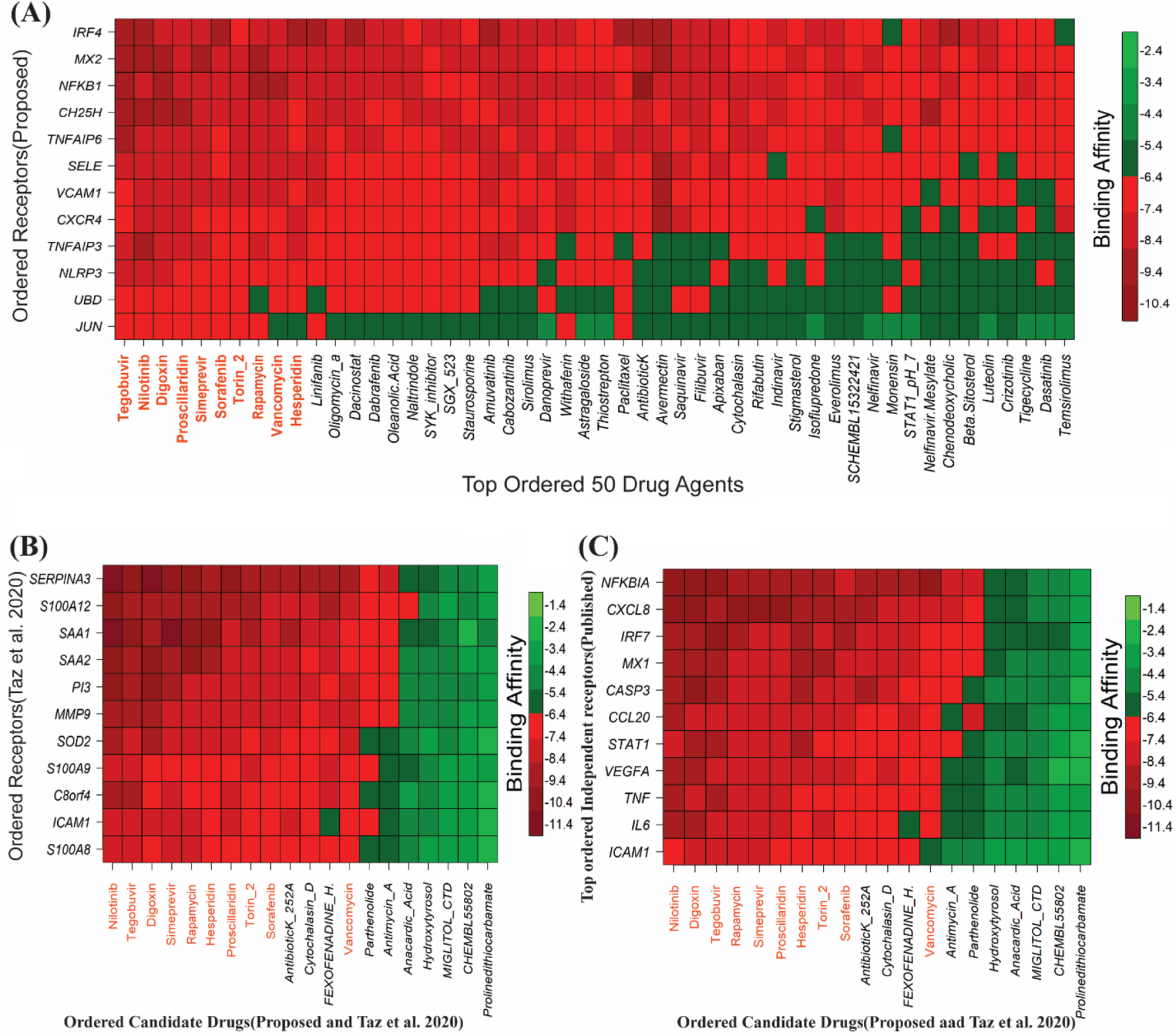
Molecular docking analysis results, where red colors indicated the strong binding affinities (**A**) Image of binding affinities based on the ordered top ranked 50 drug agents out of 185 against the ordered 13 proposed receptor proteins, (**B**) Cross-validation performance results of our proposed candidate drugs compare to the Taz et al. (2020) suggested drugs against their suggested receptors. (**C**) Cross-validation performance results of our proposed candidate drugs compare to the Taz et al. (2020) suggested drugs, against the top-ranked independent receptors published by others.

To explore cHubGs-guided candidate drug molecules by docking analysis, we downloaded 3D structure of cHubGs-mediated 10 proteins (CXCR4, IRF4, MX2, NFKB1, NLRP3, SELE, SRF, TNFAIP3, TNFAIP6, UBD) and two TFs proteins (JUN, VCAM1) as the receptor proteins from the Protein Data Bank (PDB)^51^ with source codes 2k04, 2dll, 1jnm, 4 × 0r, 2o61, 3qf2, 1esl, 1k6o, 2eqf, 1o7c, 6gf1, and 1vca, respectively. The 3D structure of CH25H target proteins were downloaded from SWISS-MODEL^53^ by using UniProt^92^ ID of O95992. The 3D structures of 185 drug agents (see Table S1(I, II)) were downloaded from PubChem database^54^ as mentioned previously. Then pairwise drug-target molecular docking analysis were carried out to calculate their binding affinity score (kcal/mol) for each pair of receptors and agents. Then, we ordered the target proteins in descending order of row average of the binding affinity matrix ***A*** = (*A*_ij_) and drug agents according to the column average to select the top-ranked drug agents as the candidate drugs (see Fig. 6A). Thus, we selected top-ranked 10 drug agents (Tegobuvir, Nilotinib, Digoxin, Proscillardin, Simeprevir, Sorafenib, Torin 2, Rapamycin, Vancomycin and Hesperidin) as candidate drug agentss with average binding affinity scores − 14.5 kcal/mol against the proposed 13 receptors. The Table 3 showed the summary results of interacting properties of top 3 candidate drugs (Tegobuvir, Nilotinib, Digoxin) with our proposed top 3 receptors (*IRF4, MX2, and NFKB1*) that produced the negatively highest binding affinity scores.

### Cross-validation (CV) of the proposed drugs

We investigated the resistance performance of our selected 10 candidate drugs compare to the Taz et al.^14^ suggested 10 drugs (mentioned introduction section) against their utilized 11 receptors as well as stat-of-art alternatives publicly available top-ranked 11 independent receptors by molecular docking. The 3D structures of 9 Taz et al. suggested receptors^14^ PI3, MMP9, SOD2, SAA1, S100A8, SERPINA3, ICAM1, S100A12 and S100A9 were retrieved from PDB with codes p0dji9, 2rel, 1l6j, 1ap6, 4ip8, q9nr00, 4ggf, 1qmn, 2oz4, 2wcb, and 1irj respectively. The 3D structures of their 2 receptors SAA2 and C8orf4 were downloaded from SWISS-MODEL^53^ using UniProt^92^ ID P0DJI9 and Q9NR00, respectively. The 3D structures of top-ranked 11 independent receptor proteins ICAM1, IRF7, MX1, NFKBIA, STAT1, IL6, TNF, CCL20, CXCL8, VEGFA and CASP3 were retrieved from PDB with codes 5mza, 2o61, 3szr, 1nfi, 1bf5, 1il6, 1tnf, 2jyo, 1ikl, 1cz8 and 1gfw, respectively. The docking results were displayed in Fig. 6B,C. We observed that our proposed candidate drugs show much better binding performance compare to the Taz et al. (2020) suggested drugs against their utilized receptors (see Fig. 6B) as well as independent receptors also (see Fig. 6C). We also investigated the binding performance of our selected 10 candidate drugs with randomly chosen 7 independent receptor proteins that were not belongs to the DEGs-set (see Fig. S1). Among them, 3D structures of POSTN, TNC, COL1A1, TACSTD2, LAMC1, COL1A2 were retrieved from PDB with ID 5WT7, 1TEN, 5CTI, 2MAE, 5XAU, and 5CTD, respectively. The “AlphaFold Protein Structure Database” was used to retrieve the 3D structures TVP23C with Uniport ID K7EK95. We observed that our suggested drug molecules do not significantly bind to those randomly selected unimportant independent receptors. Therefore, binding affinity scores (docking scores) play an important role to select the potential drug molecules.

## Discussion

**Figure 7:**
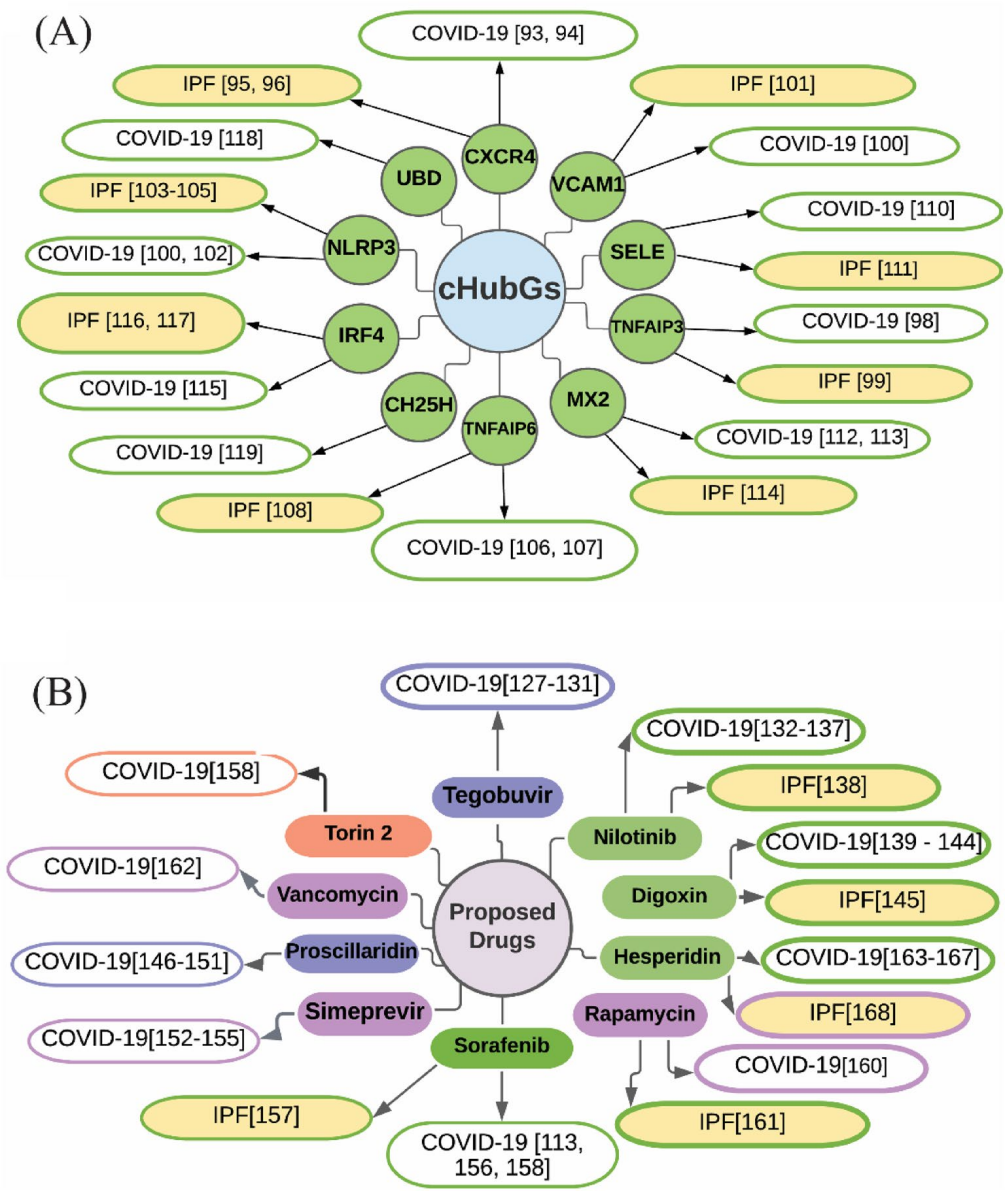
Verification of cHubGs and candidate drugs in favor of COVID-19 and IPF disease by the literature review (**A**) Verification of the proposed cHubGs: elliptical nodes with green color indicates cHubGs, and each connected network with white color node indicates the reference of COVID-19 and yellow color node indicates the reference of IPF, (**B**) Verification of the proposed candidate drugs: elliptical nodes with green color indicate FDA approved & wet-lab validated for both COVID-19 and IPF diseases, light purple color indicates FDA approved drugs, deep blue color indicate investigational drugs and light red color indicate unapproved drugs. Each connected network with white color node indicates the reference of COVID-19 and yellow color node indicates the reference of IPF.

The SARS-CoV-2 infections and IPF disease stimulate each other through the common genetic factors for which patients goes to the severe condition^14^. To investigate the genetic influence between SARS-CoV-2 infections and IPF disease, we identified 32 shared cDEGs. Among them, we detected 10 cDEGs (CXCR4, TNFAIP3, VCAM1, NLRP3, TNFAIP6, SELE, MX2, IRF4, UBD and CH25H) as cHubGs/cHubPs highlighting their functions, pathways, regulators, associated other diseases and candidate drug molecules. Some individual studies also supported the association of our proposed cHubGs with the SARS-CoV-2 infections and IPF disease as displayed in Fig. 7A. The literature review showed that CXCR4 is highly expressed in the lung cell of COVID-19 affected patients and also able to regulated the dense T cell infiltration^93^. In addition, CXCR4 signaling is considered as the key molecular regulator of different COVID-19 symptom including hypoxia and intussusceptive angiogenesis^94^. It is believed that fibrocytes, which differentiate into matrix produce myofibroblasts after transferring from the circulation into the lung, are considered as the major source of fibroblasts in IPF^95^. According to one study, the majority of circulating fibrocytes contains CXCR4^96^, and more importantly, fibrocytes are not present in healthy lungs cell^97^. TNFAIP3 overexpression could also lead to severe tissue damage and inflammation^98^. Some studies found that a number of neutrophils is significantly higher in severe SARS-CoV-2 infected patients with TNFAIP3 overexpression than the patient with no or mild infection^98^. The interaction between TNFAIP3 and glycogen synthase kinase-3β increased the C/EBP$\upbeta$ dysregulation in alveolar macrophages which considered as the major risk factor for IPF^99^. Serum Levels of VCAM-1 is significantly increased in endothelial cell of COVID-19 affected patients with mild symptom, dramatically increase in severe case and also displayed a massive reduction after antiviral treatment^100^. The gene VCAM-1 is upregulated in the IPF patients and displayed negative correlation with two major lung function such that pulmonary diffusion capacity for carbon monoxide and forced vital capacity. VCAM1 mainly found in the blood vessels and fibrotic foci of IPF affected lung with a high expression^101^. NLRP3 was found as an upregulated gene in SARS-COV2 affected patients and considered as a major biomarker to increase the cytokines level in the plasma of COVID-19 patients^100,102^. The inflammasome of NLRP3 plays a vital role in the pathology of bleomycin induced DNA damage via oxidative injury, cell death of alveolar macrophages and epithelial cells and lung injury of IPF patients^103–105^. The gene TNFAIP6(TSG-6) is a potential biomarker of mesenchymal stromal cells (MSCs) which can decrease the cytokine storm produced by SARS-CoV-2 infection. This MSCs based therapy may be potential therapeutic strategy for COVID-19 and also MSCs may be able to improve pulmonary fibrosis and lung function^106–108^. Inflammatory cytokines are considered as the major key player of SELE upregulation^109^. It has been widely suggested that the soluble form of E-selectin also known as SELE, which is released during inflammation, is a biomarker of endothelial dysfunction during COVID-19 disease^110^. The SELE gene has an important role in the pathogenesis of IPF disease^111^. An abnormal type I interferon (IFN) response is exhibited with the COVID-19 patients, which results in the deregulation of interferon-stimulated genes(ISGs) including MX2 and leads the COVID-19 severity^112,113^. The MX2 gene is significantly enriched in type I IFN signaling pathway which plays a vital role for IPF disease^114^. The IRF4 gene may regulate various altered expression of long noncoding RNAs (lncRNAs) in response to SARS-CoV-2 infection^115^ and it regulates M2 macrophages which play an important role in IPF^116,117^. The upregulations of UBD, a known inhibitor of the antiviral interferon host response, is also seen, as an increased permissiveness to the SARS-CoV-2-VSV pseudovirus^118^. Another ISGs, CH25H transforms cholesterol into 25-hydrocholesterol (25HC), and 25HC exhibits widespread anti-coronavirus behavior by preventing membrane fusion^119^. Thus, it was seen that both COVID-19 and IPF disease might be stimulated each other through the influence of cHubGs. We also investigated other diseases as the comorbidities risk factors that may stimulate both COVID-19 and IPF diseases through the influence of cHubGs by using the disease-cHubGs association studies and multivariate survival analysis of lung cancer patients with cHubGs. We found that different lung diseases including cancer^120^, Epstein-Barr virus^121^, arthritis^122^, diabetes^12^, Inflammation^122^ etc. might be the significant risk factors for severity of both COVID-19 and IPF diseases.

The cHubGs regulatory network analysis based on two databases commonly revealed two TFs proteins (JUN and NFKB1) and two miRNAs (hsa-mir-155-5p and hsa-mir-21-3p) as the transcriptional and post-transcriptional regulatory factors of cHubGs, respectively that were introduced previously in the “Results” section. Some previous studies also supported our finding that JUN is an important molecular signature for both SARS-CoV-2 infection and IPF disease^123,124^. The NFKB1 protein is considered as a potential biomarker for both SARS-COV-2 infection and IPF disease^125,126^. To investigate the common pathogenetic processes of cHubGs, we selected top five common GO terms for each of BPs, MFs and CCs, and KEGG pathways that are significantly enriched by cHubGs in at least two of three databases (see Table 3). Among them, the association of the detected BPs (*inflammatory response, response to virus, regulation of inflammatory response, response to tumor necrosis factor, response to cytokine*) with COVID-19 and IPF diseases were supported by several independent studies^60–74^. The SARS-CoV-2 infection is influenced by a severe *inflammatory response* with the release of a huge amount of *cytokine storm* known as *pro-inflammatory cytokines*. A number of studies suggested that the *cytokine storms* are associated directly with multi-organ failure, lung injury and unfavorable prognosis of SARS-CoV-2 infections^60–64^. Within the respiratory tract, persistent *inflammatory response* underlies the pathogenesis of a number of chronic pulmonary diseases including pulmonary fibrosis, asthma and chronic obstructive pulmonary disease^65^. The top five commonly enriched MFs (*ubiquitin binding, sequence-specific DNA binding,* CCR chemokine receptor binding, chemokine activity) by cHubGs, are significantly associated with both COVID-19 and IPF disease^59,75,76^. Similarly, the association of top four CCs (*tertiary granule lumen, early endosome,* extracellular space, cytosol, extracellular region) with COVID-19 and IPF disease were supported by the literature review^66,77–83^. The top five commonly enriched KEGG pathways (*TNF signaling, IL-17 signaling, NF-kappa B signaling, NOD-like receptor signaling and Cytokine-cytokine receptor interaction pathways*) are also significantly associated with COVID-19 and IPF diseases^69,84–91^. These pathways are associated with the *inflammatory response, immune response* and *the pathogen-related molecular modes recognition*. The *IL-17 signaling pathway* is related with acute respiratory distress syndrome and critical for clearing extracellular pathogens^85^. A study reported that the expression levels of different *inflammatory factors* might be increased in the SARS-CoV-2 infected patients due to the activation of *NF-kappa B signaling*^88^. The degradation of SARS-CoV-2 genome and its inhibition are influenced by the elevated *NOD-like receptor* genes^89^. On the other hand, some studies also claimed that the *TNF signaling, IL-17 signaling, NF-kappa B signaling, NOD-like receptor signaling* and *cytokine-cytokine receptor interactions* pathways were significantly enriched for IPF disease^69,90^.

**Table 3:** The 3-dimension view of strong binding interactions between targets and drugs is shown in the 4th column. The last column shows key interactions of amino acids and their binding types with potential targets.

Potential targets	Structure of lead compounds	Binding affinity (kCal/mol)	The 3d view and interactions of complex	2D view of target–ligand interaction	Interacting amino acids		
					Hydrogen bond	Hydrophobic interactions	Electrostatic
IRF4		−9.1			LYS8 LEU9	LYS8 LEU9 ARG10 ALA85 LYS63	LYS63 ARG81 ASP91
MX2		−8.6			ASN121 GLU35	LYS124 LYS125 ALA118 LEU38 LYS41 LEU24 VAL122	GLU29
NFKB1		−8.5			LYS2070 ARG2086 ASN2079	ALA2063 ALA2083 LYS2070	–

De-novo drug discovery is a time consuming, laborious and expensive procedure. In this case, drug repurposing reduces time and cost both. To explore repurposable candidate drug molecules for the treatments against SARS-CoV-2 infections with IPF disease as comorbidity risk, we considered our proposed 10 cHubGs and its regulatory 2 key TFs-proteins as the drug target receptors and performed their docking analysis with 185 meta-drug agents [see Table S1(I, II)]. Then we selected top-ranked 10 drugs (Tegobuvir^127–131^, Nilotinib^132–138^, Digoxin^139–145^, Proscillaridin^146–151^, Simeprevir^152–155^, Sorafenib^113,156–158^., Torin 2^159^, Rapamycin^160,161^, Vancomycin^162^ and Hesperidin^163–168^ as the candidate drug molecules (see Fig. 6A), where the first three drugs showed strong binding affinities with all target proteins. Then we investigated the binding performance of our proposed drug molecules compared to the Taz et al. 2020 suggested molecules by docking analysis against the Taz et al. 2020 suggested receptors^14^ and observed that our proposed drugs show much better binding performance than their suggested drugs (see Fig. 6B). Then we investigated the resistance performance of both the proposed and Taz et al. suggested drugs against the state-of-the-art alternatives top-ranked 11 independent receptors published by others for SARS-CoV-2 infections. In that case, we also observed that our proposed candidate drugs are computationally more effective compare to the TAZ et al. suggested drugs against the independent receptors (see Fig. 6C). Thus, by molecular docking approach, we observed that our proposed cHubGs-guided drug molecules show much better performance compare to the TAZ et al. (2020)^14^ suggested cDEGs-guided drug molecules. Some individual studies also recommended our proposed 10 drug molecules for the treatment against SARS-CoV-2 infections and IPF disease individually (see Fig. 7B) in which 7 molecules (Nilotinib, Digoxin, Simeprevir, Sorafenib, Rapamycin, Vancomycin and Hesperidin) are FDA approved for other diseases. Among the 7 FDA approved molecules, 4 molecules (Nilotinib, Digoxin, Sorafenib, and Hesperidin) are experimentally validated for the treatment against SARS-CoV-2 infections and IPF disease individually as discussed below.

An in vitro study reported that the drug molecule ‘Nilotinib’ (Dose: 400 mg twice daily) show a strong antiviral activity against SARS-CoV-2 and it has a safety profile that has been established for humans use and is comparatively well tolerated^134^. It also reduces the antifibrotic activity as well as pulmonary fibrosis in animal model^138^. An experimental study reported that the Digoxin drug effectively prevents over 99% of SARS-CoV-2 replication, which resulted in viral inhibitory activity at the post entry stage of the viral life cycle^139,148^. An in vitro and an in-vivo study found that the PI3K/Akt signaling pathway is inhabits by digoxin (100 nmol/L and 50 nmol/L in 12 mice model group) which may be able to regulate the activation of fibroblasts and prevent the pulmonary fibrosis that bleomycin-induced in mice^145^. The drug proscillaridin drug plays a significant role against SARS-COV-2 viral activities with 50% inhibitory concentrations values 2.04 μM^148^. An in vitro study showed that the Sorafenib drug showed antiviral activities against SAR-COV-2 by inhibiting growth factor receptor signaling pathways in non-toxic conditions^158^. The approved drugs Sorafenib one per day at a dose of 5 mg/kg body weight has strong antifibrotic activities on bleomycin induced IPF in mice model^157^. An in-vitro study found that the drug molecule ‘simeprevir’ significantly decreases the SARS-CoV-2 viral load^152^. In a double-blind placebo-controlled trial, the drug ‘Hesperidin 1000 mg daily may help decrease fever, cough, shortness of breath, or anosmia symptoms of nonvaccinated COVID-19 infected patients^169^. In histopathological, biochemical, and micro-CT analyses on rat, Hesperidin successfully reduced the severity of lung injury caused by BLC, a model for IPF^168^. Therefore, the findings of this study might be useful resources for simultaneously diagnosis and therapies of the SARS-CoV-2 infections and IPF diseases.

## Conclusion

In this article, we detected 10 common hub-genes (cHubGs) that can differentiate both COVID-19 and IPF diseases from the control samples based on their expression patterns by using the integrated bioinformatics approaches. We observed that our proposed cHubGs (CXCR4, TNFAIP3, VCAM1, NLRP3, TNFAIP6, SELE, MX2, IRF4, UBD and CH25H) were more representative of common differentially expressed genes (cDEGs) than the previously suggested cHubGs by others. The cHubGs regulatory network analysis commonly detected SARS-CoV-2 and IPF diseases causing two crucial transcriptional (JUN and NFKB1) and post-transcriptional (hsa-mir-155-5p and hsa-mir-21-3p) regulators of cHubGs based on three independent databases JASPAR, TarBase and RegNetwork. The cHubGs-set enrichment analysis commonly detected some crucial GO-terms (BPs, CCs, and MFs) and KEGG pathways that are significantly associated with the development mechanisms of SARS-CoV-2 and IPF diseases from three independent databases Enrichr, DAVID and GeneCodis. Then we recommended our proposed cHubGs-guided 10 candidate drugs for the treatment against the SAR-CoV-2 infections with IPF disease as co-existing risk factor. We observed that our suggested drugs are computationally more efficient compare to the previously suggested drug molecules for the treatment against the SAR-CoV-2 infections with IPF disease as comorbidity risk factor. Among our proposed ten drug molecules, seven molecules (Nilotinib, Digoxin, Simeprevir, Sorafenib, Rapamycin, Vancomycin and Hesperidin) are FDA approved for other diseases, where four molecules (Nilotinib, Digoxin, Sorafenib, and Hesperidin) are experimentally validated for the treatment against SARS-CoV-2 infections and IPF disease individually. Thus, our proposed molecular biomarkers and candidate drug molecules in this study has a merit for diagnosis and therapies of SAR-CoV-2 infections with IPF disease as comorbidity risk.

## Supplementary Information

Supplementary Information.

## Data Availability

The datasets of RNA-Seq profiles on SARS-CoV-2 infections and IPF diseases that were analyzed in this study are collected from the National Center for Biotechnology Information (NCBI) Gene Expression Omnibus (GEO) open access database, where datasets are freely available (http://www.ncbi.nlm.nih.gov/geo/). The RNA-Seq profiles dataset on SARS-CoV-2 infected patients were downloaded from GPL18573 platform of Illumina NextGen 500 (Homo sapiens) with the GEO accession numbers GSE147507 published by Blanco-Melo et al.^21^. On the hand, the RNA-Seq profiles dataset on IPF patients were downloaded from GPL11154 platform of Illumina HiSeq 2000 (Homo sapiens) with the GEO accession numbers GSE52463 published by Nance et al.^22^.
