# Supplementary material for: Bioinformatics-based investigation on the genetic influence between SARS-CoV-2 infections and idiopathic pulmonary fibrosis (IPF) diseases, and drug repurposing
Source: Sci Rep. 2023 Mar 22;13:4685. doi: 10.1038/s41598-023-31276-6 (PMC10031699; doi:10.1038/s41598-023-31276-6)
Supplement: Supplementary file 1 — Supplementary Information. [file 41598_2023_31276_MOESM1_ESM.docx]

**Supplementary File**

**Bioinformatics-based investigation on the genetic influence between SARS-CoV-2 infections and idiopathic pulmonary fibrosis (IPF) diseases, and drug repurposing**

Md. Ariful Islam^1^, Md. Kaderi Kibria^1^, Md. Bayazid Hossen^1^, Md. Selim Reza^1^, Samme Amena Tasmia^1^, Khanis Farhana Tuly^1^, Md. Parvez Mosharof^1^, Syed Rashel Kabir^2^, Md. Hadiul Kabir^1^ and Md. Nurul Haque Mollah^1^*

^1^Bioinformatics Lab, Department of Statistics, University of Rajshahi, Rajshahi-6205, Bangladesh

^2^Department of Biochemistry and Molecular Biology, University of Rajshahi, Rajshahi-6205, Bangladesh.

Email id of authors are respectively: [ariful.stat.bio@gmail.com](mailto:ariful.stat.bio@gmail.com); [kibriastat15@gmail.com](mailto:kibriastat15@gmail.com); [bayazid.stat@gmail.com](mailto:bayazid.stat@gmail.com);

[selim.ru4778@gmail.com](mailto:selim.ru4778@gmail.com); [jusi.stat14@gmail.com](mailto:jusi.stat14@gmail.com); [farhanatuly119@gmail.com](mailto:farhanatuly119@gmail.com); [parvezstatru@gmail.com](mailto:parvezstatru@gmail.com); [rashelkabir@ru.ac.bd](mailto:rashelkabir@ru.ac.bd); [hk@ru.ac.bd](mailto:hk@ru.ac.bd); [mollah.stat.bio@ru.ac.bd](mailto:mollah.stat.bio@ru.ac.bd).

***Corresponding Author:** Md. Nurul Haque Mollah (E-mail: [mollah.stat.bio@ru.ac.bd](mailto:mollah.stat.bio@ru.ac.bd))


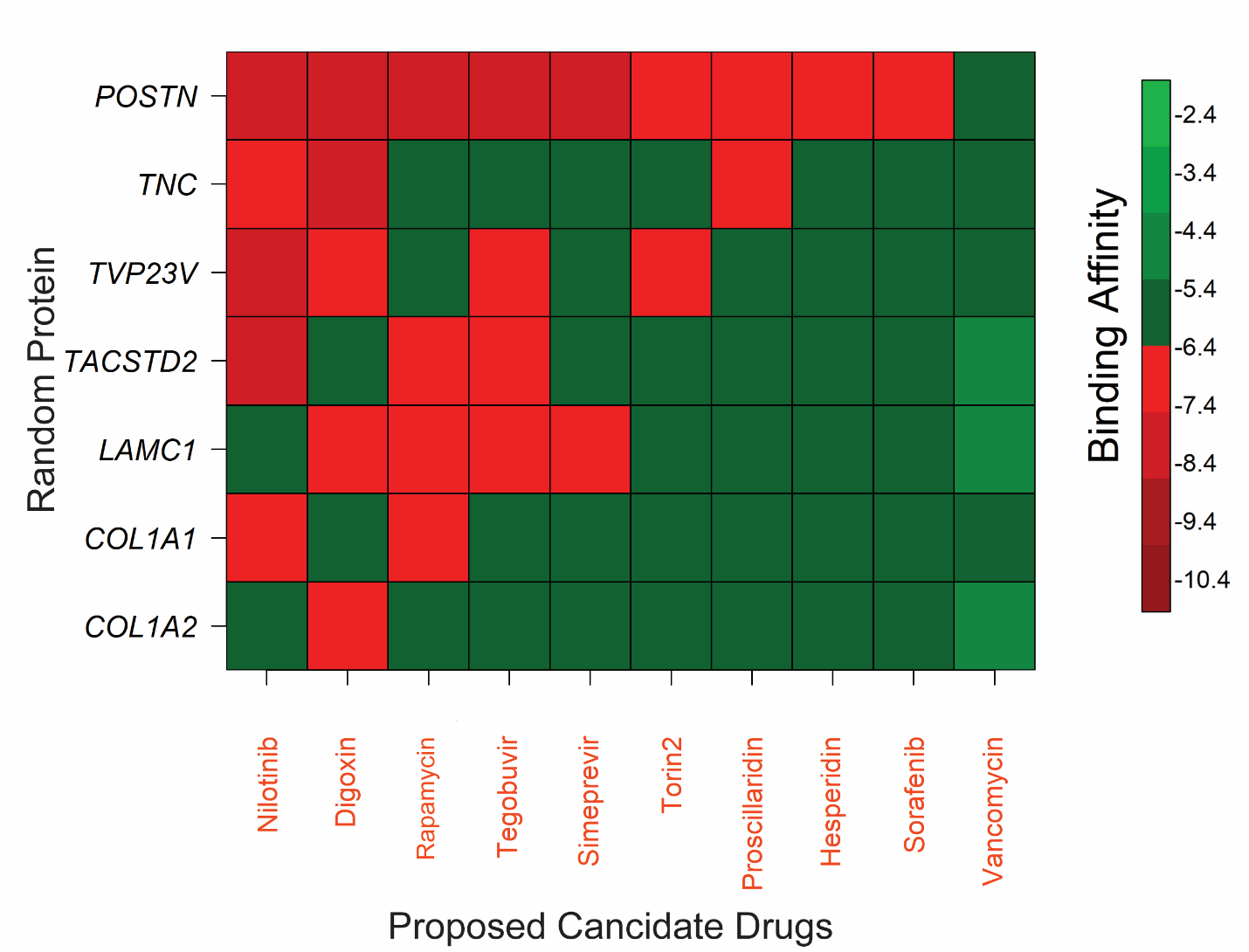


**Figure S1:** Molecular docking analysis results to preform cross-validation of our proposed candidate drugs with random protein, where red colors indicated the strong binding affinities.

.

| **Table S1(I): SARS-COV-2 3CL protease-guided 90 drugs including FDA authorized anti-viral drugs** | |
| --- | --- |
| **Published Articles** | **Suggested Drug** |
| Beck et al. ^1^ | 5-nonyloxytryptamine, Abacavir sulfate, Abacavir, Acetylcholine Chloride, Acyclovir, Adefovir Dipivoxil, Amprenavir (agenerase), Apixaban, Asunaprevir (BMS-650032), Atazanavir sulfate (BMS-232632-05), Atazanavir, Atropine, avermectin, Batimastat, Boceprevir, bosutinib, Cidofovir, Cyclosporine, dacinostat, Daclatasvir (BMS-790052), danoprevir, Daptomycin, Darunavir, demecarium, Difloxacin HCl, dinoprostone, efavirenz, elvitegravir, Entecavir Hydrate, entecavir, eprosartan, Etomidate, everolimus, Famciclovir, foxy-5, Ganciclovir, indinavir, ivermectin, Leuprolide Acetate, lisuride, lopinavir, Methscopolamine, mupirocin, naltrindole, Nelfinavir Mesylate, nelfinavir, nevirapine, Octreotide acetate, oligomycin-a, Oseltamivir acid, Oseltamivir phosphate, Oseltamivir, Otilonium Bromide, Penciclovir, Peramivir Trihydrate, Peramivir, Pimecrolimus, prostaglandin, Radotinib(IY-5511), raltegravir, Rapamycin (Sirolimus), Remdesivir, ribavirin, Rifabutin, Rilpivirine, Ritonavir, Rupatadine Fumarate, Saquinavir mesylate, saquinavir, saracatinib, scopolamine, Sildenafil Citrate, sirolimus, somatostatin, Tacrolimus (FK506), Telaprevir (VX-950), temsirolimus, Tenofovir Disoproxil Fumarate, tenofovir, thiostrepton, Tigecycline, Tiotropium Bromide, torin-2, trichostatin-a, Valaciclovir HCl, valaciclovir, Valganciclovir HCl, Zanamivir, zolmitriptan, PHA-665752 |

| **Table S1(II):** Transcriptome-guided 87 meta-drug agents associated with SARS-CoV-2 infections collected by literature review**.** | |
| --- | --- |
| Kartikay Prasad et al. 2020^2^ | Mitomycin-C, Imiquimod, Polyinosinic:polycytidylic acid (poly I:C), S-carbamidomethylcysteine (Cysteine-S-acetamide), Vanadium oxide and MgATP. |
| Gurudeeban Selvaraj et al. 2021^3^ | Wortmannin |
| Ruan et al., 2021^4^ | Nilotinib, Saquinavir, Tipranavir, Lonafarnib, Tegobuvir, Olysio, Filibuvir, and Cepharanthine |
| Taz et al. 2020^5^ | MIGLITOL CTD 00002031, CHEMBL55802 CTD 00003118, Hesperidin CTD 00006087, Cytochalasin D CTD 00007076, Prolinedithiocarbamate CTD 00002658, Parthenolide CTD 00000087, FEXOFENADINE HYDROCHLORIDE CTD 00003191, Hydroxytyrosol CTD 00000267, Antimycin A CTD 00005427, Anacardic acid C15:3 CTD 00003117, |
| Moni et al. 2020^6^ | Cytochalasin D, 1′-acetoxychavicol acetate, Atorvastatin, Proline dithiocarbamate, Dicumarol, Oleanolic acid. |
| Islam et al. 2020^7^ | SYK-inhibitor, Radicicol, Dabrafenib, AT-7519, Dasatinib, Lovastatin, Thiostrepton, Linifanib, JNK–IN–5A, Withaferin-A. |
| Ge C et al 2020^8^ | Astragaloside IV |
| Aishwarya et al. 2020^9^ | F-1566-0341, Digoxin, Proscillaridin, Linifanib |
| Tao et. al., 2020^10^ | Quercetin, Kaempferol, Beta-sitosterol, Stigmasterol, Isorhamnetin, Baicalein, Naringenin, Formononetin |
| Han et. al, 2020 ^11^ | Quercetin, Luteolin |
| Li Zhonglin et al, 2020^12^ | Podophyllotoxin, Amantadine, Thioperamide, Monensin, Vancomycin, Etiocholanolone, Acyclovir, Isoflupredone, Heptaminol,Chenodeoxycholic acid, Podophyllotoxin, Atractyloside, Adiphenine, Monensin, Lisuride |
| Fangzhou Liu_2021^13^ | Matrine |
| Zulkar Nain et al. 2020^14^ | Antibiotic K-252A,Cabozantinib, Amuvatinib, Crizotinib, SGX-523, [888719-03-7](https://www.ncbi.nlm.nih.gov/pcsubstance/?term=%22888719-03-7%22%5bCompleteSynonym%5d%20AND%2021081761%5bStandardizedCID%5d), CHEMBL527066, [CHEMBL503090](https://www.ncbi.nlm.nih.gov/pcsubstance/?term=%22CHEMBL503090%22%5bCompleteSynonym%5d%20AND%2011560856%5bStandardizedCID%5d), SCHEMBL15322421, CHEMBL462712, [rac-crizotinib](https://www.ncbi.nlm.nih.gov/pcsubstance/?term=%22rac-crizotinib%22%5bCompleteSynonym%5d%20AND%2011597571%5bStandardizedCID%5d), [CHEMBL561660](https://www.ncbi.nlm.nih.gov/pcsubstance/?term=%22CHEMBL561660%22%5bCompleteSynonym%5d%20AND%2025229537%5bStandardizedCID%5d), Crizotinib, Cabozantinib. |
| Suresh Kumar _et al. 2020 ^15^ | Chloroquine, lenalidomide, Penicillin, Pentoxifylline, Thalidome, Sorafenib,  Paclitaxel, Rapamycin, Cortisol, Statins |
| Yi-Wei Zhu et al. 2020 ^16^ | Quercetin, Kaempferol, bsitosterol, Isorhamnetin, Naringenin, Luteolin, (þ)-catechin, Delphinidin, aloe-Emodin, Baicalein and Irisolidone |
| Zhen-Zhen^17^ | berberine/NIT-X |

| **Table S1(III):** List of different lists of hub genes (HubGs) for SARS-CoV-2 infections collected by literature review**.** | | | |
| --- | --- | --- | --- |
| **Articles** | **Hub Genes (HubGs)** | **Common hub genes with at least 3 articles** | **Common hub genes with at least 4 articles** |
| **Xie et al. 2020** ^18^ | CXCL1, CXCL2, TNF, NFKBIA, CSF2, TNFAIP3, IL6, CXCL3, CCL20, ICAM1 | IL6, TNF, CCL20, CXCL8, VEGFA, ICAM1, IRF7, MX1, NFKBIA, STAT1, CASP3 | IL6, TNF, VEGFA |
| **Oh JH et al. 2020** ^19^ | GATA4, ID2, MAFA, NOX4, PTBP1, SMAD3, TUBB1, WWOX |  |  |
| **Vastrad et al. 2020** ^20^ | TP53, HRAS, CTNNB1, FYN, ABL1, STAT3, STAT1, JAK2, C1QBP, XBP1, BST2, CD99, IFI35, MAPK11, RELA, LCK, KIT, EGR1, IL20, ILF3, CASP3, IL19, ATG7, GPI, S1PR1 |  |  |
| **Prasad et al. 2020** ^2^ | STAT1, IRF7, IFIH1, MX1, ISG15, IFIT3, OAS2, DDX58, IRF9, IFIT1,  OAS1, OAS3, DDX60, OASL, IFIT2 |  |  |
| **Selvaraj et al. 2021** ^3^ | MYC, HDAC9, NCOA3, CEBPB, VEGFA, BCL3, SMAD3, SMURF1, KLHL12, CBL, ERBB4, CRKL |  |  |
| **Satu et al. 2021**^21^ | MARCO, VCAN, ACTB, LGALS1, HMOX1, TIMP1, OAS2, GAPDH, MSH3, FN1, NPC2, JUND, GPNMB, SYTL2, CASP1, S100A8, MYO10, IGFBP3, APCDD1, COL6A3, FABP5, PRDX3, CLEC1B, DDIT4, CXCL10, CXCL8 |  |  |
| **Taz et al. 2021** ^22^ | VEGFA, AKT1, MMP9, ICAM1, CD44 |  |  |
| **Moni et al. 2020** ^6^ | MX1, IRF7, BST2 |  |  |
| **Islam et al. 2020** ^7^ | BIRC3, ICAM1, IRAK2, MAP3K8, S100A8, SOCS3, STAT5A, TNF, TNFAIP3, TNIP1 |  |  |
| **Zhou et al. 2020** ^23^ | JUN, XPO1, NPM1, HNRNPA1 |  |  |
| **Ge et al. 2020** ^8^ | MMP13, NLRP3, GBP1, ADORA2A, PTAFR, TNF, MLNR, IL1B, NFKBIA, ADRB2, IL6 |  |  |
| **Aishwarya et al. 2020** ^9^ | IGF2, HINT1, MAPK10, SGCE, HDAC5, SGCA, SGCB, CFD, ITSN1, EHMT2, CLU, ISLR, PGM5, ANK2, HDAC9, SYT11, MDH1, SCCPDH, SIRT6, DTNA, FN1, ARRB1, MAGED2, TEX264, VEGFC, HK2, TXNL4A, SLC16A3, NUDT21, TRA2B, HNRNPA1, CDC40, THOC1, PFKFB3 |  |  |
| **Saxena, et al 2020** ^24^ | STAP1, CASP5, FDCSP, CARD17, ST20, AKR1B10, CLC, KCNJ2-AS1, RNASE2 , FLG |  |  |
| **Tao et al. 2020** ^10^ | MAPK3, MAPK8, TP53, CASP3, IL6, TNF, MAPK1, CCL2, PTGS2 |  |  |
| **Zhang et. al. 2020** ^25^ | CXCL10, ISG15, DDX58, MX2, OASL, STAT1, RSAD2, MX1, IRF7, OAS1 |  |  |
| **Han L et. al, 2020** ^11^ | IL6 , TNF ,IL10, MAPK8,MAPK3,CXCL8,CASP3,PTGS2, TP53, MAPK1 |  |  |
| **Wang et al. 2020** ^26^ | CXCL8, CXCL1, CXCL2, CCL20, CSF2 |  |  |
| **Gu et al. 2020** ^27^ | NFKBIA, C3, CCL20, BCL2A1, BID |  |  |
| **Nan et al. 2021** ^28^ | ALB, CXCL8, FGF2, IL6, INS, MMP2, MMP9, PTGS2, STAT3, VEGFA |  |  |
| **Gu et al. 2020** ^29^ | CDC20, NCBP1, POLR2D, DYNLL1, FBXW5, LRRC41, FBXO21, FBXW9, FBXO44, FBXO6 |  |  |
| **Sardar et al. 2020** ^30^ | HMOX1, DNMT1, PLAT, GDF1, ITGB1 |  |  |
| **Gu et al. 2020** ^31^ | FLOC, DYNLL1, FBXL3, FBXW11, FBXO27, FBXO44, FBXO32, FBXO31, FBXO9, CUL2 |  |  |

**Table S2:** Selection procedure of proposed common hub genes of String PPI (cHubGs).

|  | Degree | Betweenness | BottleNeck | Closeness | Stress | cHubGs |
| --- | --- | --- | --- | --- | --- | --- |
| Top 3 | CXCR4,  TNFAIP3, VCAM1. | TNFAIP3,  VCAM1,  CH25H. | TNFAIP3, CXCR4, IRF4. | TNFAIP3, CXCR4,  VCAM1. | TNFAIP3, CXCR4, VCAM1. | CXCR4, TNFAIP3, VCAM1, CH25H, IRF4. |
| Top 5 | CXCR4  TNFAIP3, VCAM1,  NLRP3, TNFAIP6. | TNFAIP3,  VCAM1,  CH25H,  CXCR4,  NLRP3. | TNFAIP3, CXCR4, IRF4,  SELE, NLRP3. | TNFAIP3, CXCR4,  VCAM1,  NLRP3,  MX2. | TNFAIP3, CXCR4, VCAM1,  NLRP3,  TNFAIP6. | CXCR4, TNFAIP3, VCAM1, NLRP3, TNFAIP6, CH25H, SELE, IRF4, MX2. |
| Top 7 | CXCR4  TNFAIP3, VCAM1,  NLRP3, TNFAIP6,  SELE,  MX2. | TNFAIP3,  VCAM1,  CH25H,  CXCR4,  NLRP3,  TNFAIP6,  MX2. | TNFAIP3, CXCR4, IRF4,  SELE, NLRP3, UBD,  CH25H. | TNFAIP3, CXCR4,  VCAM1,  NLRP3,  MX2,  TNFAIP6,  SELE. | TNFAIP3, CXCR4, VCAM1,  NLRP3,  TNFAIP6,  MX2,  SELE. | CXCR4, TNFAIP3, VCAM1, NLRP3, TNFAIP6, SELE, MX2, CH25H, IRF4, UBD. |

**Table S3:** The list of significantly (p-value<0.001) comorbidities associated with cHubGs.

| **Diseases** | **P-value** | **cHubGs** |
| --- | --- | --- |
| Epstein-Barr Virus Infections | 4.86E-10 | VCAM1;TNFAIP6;IRF4;TNFAIP3;CXCR4;SELE |
| Multiple Sclerosis, Acute Fulminating | 4.24E-09 | VCAM1;TNFAIP3;NLRP3;SELE |
| Juvenile arthritis | 2.63E-07 | TNFAIP6;TNFAIP3;NLRP3;CXCR4;SELE |
| Inflammation | 1.02E-06 | VCAM1;TNFAIP6;TNFAIP3;NLRP3;SELE |
| Eczema | 1.35E-06 | VCAM1;UBD;NLRP3;CXCR4;SELE |
| Inflammatory disorder | 1.65E-06 | VCAM1;NLRP3;CXCR4;SELE |
| Dermatitis, Atopic | 1.85E-06 | VCAM1;UBD;NLRP3;CXCR4;SELE |
| Thrombocytopenia due to platelet alloimmunization | 2.08E-06 | TNFAIP3;NLRP3;CXCR4 |
| Myocardial Infarction | 2.22E-06 | VCAM1;TNFAIP6;UBD;NLRP3;CXCR4;SELE |
| Degenerative polyarthritis | 2.36E-06 | VCAM1;TNFAIP6;IRF4;NLRP3;CXCR4;SELE |
| Lupus Nephritis | 2.36E-06 | VCAM1;TNFAIP3;NLRP3;SELE |
| Lymphoma, Non-Hodgkin | 3.14E-06 | VCAM1;IRF4;NLRP3;CXCR4;SELE |
| Hypercholesterolemia | 4.01E-06 | VCAM1;NLRP3;CXCR4;SELE |
| Multiple Sclerosis | 4.89E-06 | CH25H;VCAM1;TNFAIP3;NLRP3;CXCR4;SELE |
| Arthritis | 6.75E-06 | VCAM1;TNFAIP6;TNFAIP3;NLRP3;CXCR4 |
| Diabetes | 1.08E-05 | VCAM1;UBD;TNFAIP3;NLRP3;CXCR4;SELE |
| Hereditary Autoinflammatory Diseases | 1.23E-05 | TNFAIP3;NLRP3 |
| Encephalomyelitis | 1.74E-05 | CH25H;IRF4;NLRP3;CXCR4 |
| Cerebrovascular accident | 1.77E-05 | VCAM1;IRF4;NLRP3;CXCR4;SELE |
| Diabetes Mellitus | 2.92E-05 | VCAM1;UBD;TNFAIP3;NLRP3;CXCR4;SELE |
| Peritonitis | 2.97E-05 | VCAM1;NLRP3;SELE |
| Juvenile rheumatoid arthritis | 3.19E-05 | NLRP3;CXCR4;SELE |
| Virus Diseases | 3.32E-05 | VCAM1;IRF4;NLRP3;CXCR4;SELE |
| melanoma | 3.55E-05 | VCAM1;IRF4;MX2;UBD;NLRP3;CXCR4;SELE |
| Periodontal Diseases | 3.64E-05 | NLRP3;CXCR4;SELE |
| Crohn Disease | 4.06E-05 | IRF4;UBD;TNFAIP3;NLRP3;CXCR4 |
| Ulcerative Colitis | 4.12E-05 | VCAM1;IRF4;TNFAIP3;NLRP3;CXCR4 |
| Encephalitis | 4.23E-05 | VCAM1;NLRP3;CXCR4 |
| Rheumatoid Nodule | 4.70E-05 | VCAM1;SELE |
| Chronic venous insufficiency | 4.70E-05 | VCAM1;SELE |
| Aneurysm | 4.97E-05 | VCAM1;NLRP3;SELE |
| Diabetes Mellitus, Insulin-Dependent | 5.09E-05 | VCAM1;TNFAIP6;TNFAIP3;NLRP3;SELE |
| Cluster Headache | 5.17E-05 | VCAM1;SELE |
| Bone Cysts, Aneurysmal | 5.66E-05 | TNFAIP3;NLRP3 |
| Granulomatous Slack Skin | 5.66E-05 | IRF4;TNFAIP3 |
| Coronary Artery Disease | 5.76E-05 | VCAM1;TNFAIP3;NLRP3;CXCR4;SELE |
| Inflammatory dermatosis | 6.11E-05 | VCAM1;NLRP3;CXCR4 |
| Allergic rhinitis (disorder) | 6.11E-05 | IRF4;NLRP3;CXCR4 |
| Lymphomatoid Papulosis | 6.71E-05 | IRF4;CXCR4 |
| Infection | 6.75E-05 | VCAM1;NLRP3;CXCR4;SELE |
| Adult T-Cell Lymphoma/Leukemia | 7.19E-05 | VCAM1;IRF4;NLRP3;CXCR4 |
| Dengue Fever | 7.81E-05 | VCAM1;NLRP3;SELE |
| Idiopathic Interstitial Pneumonias | 7.85E-05 | CXCR4;SELE |
| Immune thrombocytopenic purpura | 8.63E-05 | TNFAIP3;NLRP3;CXCR4 |
| Rheumatoid Arthritis | 8.92E-05 | VCAM1;IRF4;TNFAIP3;NLRP3;CXCR4;SELE |
| Sepsis | 8.95E-05 | VCAM1;NLRP3;CXCR4;SELE |
| Anthracosis | 9.07E-05 | NLRP3;SELE |
| Arteriosclerosis | 9.41E-05 | CH25H;VCAM1;NLRP3;CXCR4;SELE |
| Lupus Erythematosus, Systemic | 1.05E-04 | VCAM1;TNFAIP3;NLRP3;CXCR4;SELE |
| Periodic Fever Syndrome | 1.11E-04 | TNFAIP3;NLRP3 |
| Systemic Scleroderma | 1.13E-04 | TNFAIP3;NLRP3;CXCR4;SELE |
| Colorectal Carcinoma | 1.15E-04 | VCAM1;IRF4;UBD;TNFAIP3;NLRP3;CXCR4;SELE |
| Atherosclerosis | 1.15E-04 | CH25H;VCAM1;NLRP3;CXCR4;SELE |
| Superinfection | 1.18E-04 | NLRP3;CXCR4 |
| Glomerulonephritis | 1.30E-04 | VCAM1;CXCR4;SELE |
| Lymphoproliferative Disorders | 1.39E-04 | IRF4;TNFAIP3;CXCR4 |
| Respiratory syncytial virus (RSV) infection in conditions classified elsewhere and of unspecified site | 1.41E-04 | VCAM1;NLRP3;SELE |
| Brucellosis | 1.48E-04 | NLRP3;SELE |
| Liver diseases | 1.52E-04 | VCAM1;NLRP3;CXCR4;SELE |
| Colitis | 1.53E-04 | VCAM1;IRF4;NLRP3;SELE |
| Painful Bladder Syndrome | 1.57E-04 | CXCR4;SELE |
| Stage IV Ovarian Carcinoma | 1.65E-04 | VCAM1;CXCR4 |
| Hamman-Rich syndrome | 1.76E-04 | VCAM1;NLRP3;CXCR4 |
| Lymphoma | 2.26E-04 | VCAM1;IRF4;TNFAIP3;NLRP3;CXCR4 |
| Acute Coronary Syndrome | 2.30E-04 | VCAM1;NLRP3;SELE |
| Asthma | 2.31E-04 | VCAM1;IRF4;TNFAIP3;NLRP3;SELE |
| Behcet Syndrome | 2.38E-04 | VCAM1;TNFAIP3;NLRP3 |
| Sjogren's Syndrome | 2.44E-04 | TNFAIP3;NLRP3;CXCR4 |
| Lymphoma, T-Cell, Cutaneous | 2.44E-04 | IRF4;TNFAIP3;CXCR4 |
| Colorectal Cancer | 2.49E-04 | VCAM1;IRF4;UBD;TNFAIP3;NLRP3;CXCR4;SELE |
| Endothelial dysfunction | 2.49E-04 | VCAM1;NLRP3;SELE |
| Fabry Disease | 2.51E-04 | VCAM1;SELE |
| Thromboangiitis Obliterans | 2.61E-04 | VCAM1;SELE |
| Chronic inflammatory disorder | 2.72E-04 | NLRP3;SELE |
| Active tuberculosis | 2.72E-04 | NLRP3;CXCR4 |
| B-Cell Lymphomas | 3.10E-04 | VCAM1;IRF4;TNFAIP3;CXCR4 |
| Leukemogenesis | 3.10E-04 | IRF4;TNFAIP3;CXCR4;SELE |
| Aortic Aneurysm, Abdominal | 3.29E-04 | VCAM1;NLRP3;CXCR4 |
| Anoxia | 3.29E-04 | VCAM1;TNFAIP3;CXCR4 |
| Gingivitis | 3.29E-04 | NLRP3;SELE |
| Primary central nervous system lymphoma | 3.29E-04 | IRF4;TNFAIP3 |
| Severe Dengue | 3.66E-04 | VCAM1;NLRP3 |
| Bullous pemphigoid | 3.92E-04 | VCAM1;SELE |
| Celiac Disease | 4.16E-04 | TNFAIP3;NLRP3;SELE |
| Lichen Planus, Oral | 4.32E-04 | VCAM1;SELE |
| Neutrophilia (disorder) | 4.32E-04 | CXCR4;SELE |
| Bronchiolitis Obliterans | 4.46E-04 | CXCR4;SELE |
| Systemic Inflammatory Response Syndrome | 4.60E-04 | TNFAIP3;SELE |
| Paroxysmal atrial fibrillation | 4.74E-04 | VCAM1;SELE |
| Idiopathic Inflammatory Myopathies | 4.89E-04 | TNFAIP3;CXCR4 |
| Secondary malignant neoplasm of bone | 4.90E-04 | VCAM1;CXCR4;SELE |
| Malignant mesothelioma | 5.12E-04 | VCAM1;NLRP3;CXCR4 |
| Pneumonitis | 5.30E-04 | VCAM1;NLRP3;CXCR4 |
| Tracheal Diseases | 5.49E-04 | VCAM1;NLRP3 |
| Hepatitis, Alcoholic | 5.80E-04 | UBD;CXCR4 |
| Marginal Zone B-Cell Lymphoma | 5.96E-04 | VCAM1;TNFAIP3 |
| Lymphoma, Follicular | 6.27E-04 | IRF4;TNFAIP3;CXCR4 |
| Autoimmune thrombocytopenia | 6.29E-04 | TNFAIP3;NLRP3 |
| Presenile dementia | 6.42E-04 | VCAM1;CXCR4;SELE |
| Leukemia, T-Cell | 6.52E-04 | VCAM1;IRF4;CXCR4 |
| Precursor B-cell lymphoblastic leukemia | 6.68E-04 | IRF4;CXCR4;SELE |
| Diabetes Mellitus, Non-Insulin-Dependent | 7.15E-04 | VCAM1;TNFAIP3;NLRP3;CXCR4;SELE |
| Inflammatory Bowel Diseases | 7.20E-04 | VCAM1;IRF4;NLRP3;CXCR4 |
| Coronary heart disease | 7.26E-04 | TNFAIP3;NLRP3;CXCR4;SELE |
| Idiopathic Pulmonary Fibrosis | 7.34E-04 | VCAM1;NLRP3;CXCR4 |
| Chronic Kidney Diseases | 7.39E-04 | VCAM1;NLRP3;CXCR4 |
| Lymphoma, Large-Cell, Follicular | 7.49E-04 | TNFAIP3;CXCR4 |
| Neoplasm Metastasis | 7.57E-04 | CH25H;VCAM1;IRF4;UBD;TNFAIP3;CXCR4;SELE |
| Malaria, Cerebral | 8.04E-04 | VCAM1;SELE |
| Lung diseases | 8.15E-04 | TNFAIP6;NLRP3;CXCR4 |
| Ischemic stroke | 8.21E-04 | VCAM1;IRF4;SELE |
| Vascular Diseases | 8.64E-04 | VCAM1;CXCR4;SELE |
| Periodontitis, Juvenile | 8.80E-04 | VCAM1;SELE |
| Dermatologic disorders | 8.96E-04 | VCAM1;TNFAIP6;NLRP3 |
| Pulmonary Fibrosis | 8.98E-04 | NLRP3;CXCR4;SELE |

**References**

1. Beck, B. R., Shin, B., Choi, Y., Park, S. & Kang, K. Predicting commercially available antiviral drugs that may act on the novel coronavirus (SARS-CoV-2) through a drug-target interaction deep learning model. *Comput. Struct. Biotechnol. J.* **18**, (2020).

2. Prasad, K. *et al.* Targeting hub genes and pathways of innate immune response in COVID-19: A network biology perspective. *Int. J. Biol. Macromol.* **163**, (2020).

3. Selvaraj, G., Kaliamurthi, S., Peslherbe, G. H. & Wei, D.-Q. Identifying potential drug targets and candidate drugs for COVID-19: biological networks and structural modeling approaches. *F1000Research* **10**, 127 (2021).

4. Ruan, Z. *et al.* SARS-CoV-2 and SARS-CoV: Virtual screening of potential inhibitors targeting RNA-dependent RNA polymerase activity (NSP12). *J. Med. Virol.* **93**, (2021).

5. Taz, T. A. *et al.* Network-based identification genetic effect of SARS-CoV-2 infections to Idiopathic pulmonary fibrosis (IPF) patients. *Brief. Bioinform.* **00**, 1–13 (2020).

6. Moni, M. A., Quinn, J. M. W., Sinmaz, N. & Summers, M. A. Gene expression profiling of SARS-CoV-2 infections reveal distinct primary lung cell and systemic immune infection responses that identify pathways relevant in COVID-19 disease. *Brief. Bioinform.* **00**, 1–14 (2020).

7. Islam, T. *et al.* Integrative transcriptomics analysis of lung epithelial cells and identification of repurposable drug candidates for COVID-19. *Eur. J. Pharmacol.* **887**, 173594 (2020).

8. Ge, C. & He, Y. In Silico Prediction of Molecular Targets of Astragaloside IV for Alleviation of COVID-19 Hyperinflammation by Systems Network Pharmacology and Bioinformatic Gene Expression Analysis. *Front. Pharmacol.* **11**, (2020).

9. Aishwarya, S., Gunasekaran, K. & Margret, A. A. Computational gene expression profiling in the exploration of biomarkers, non-coding functional RNAs and drug perturbagens for COVID-19. *J. Biomol. Struct. Dyn.* **0**, 1–16 (2020).

10. Tao, Q. *et al.* Network pharmacology and molecular docking analysis on molecular targets and mechanisms of Huashi Baidu formula in the treatment of COVID-19. *Drug Dev. Ind. Pharm.* **46**, 1–9 (2020).

11. Han, L. *et al.* Potential mechanism prediction of Cold-Damp Plague Formula against COVID-19 via network pharmacology analysis and molecular docking. *Chinese Med. (United Kingdom)* **15**, (2020).

12. Li, Z. & Yang, L. Underlying Mechanisms and Candidate Drugs for COVID-19 Based on the Connectivity Map Database. *Front. Genet.* **11**, (2020).

13. Liu, F. *et al.* Study on mechanism of matrine in treatment of COVID-19 combined with liver injury by network pharmacology and molecular docking technology. *Drug Deliv.* **28**, (2021).

14. Nain, Z. *et al.* Pathogenetic profiling of COVID-19 and SARS-like viruses. *Brief. Bioinform.* **22**, (2021).

15. Kumar, S. COVID-19: A drug repurposing and biomarker identification by using comprehensive gene-disease associations through protein-protein interaction network analysis. *Preprints* (2020) doi:10.20944/preprints202003.0440.v1.

16. Zhu, Y. W. *et al.* Analyzing the potential therapeutic mechanism of Huashi Baidu Decoction on severe COVID-19 through integrating network pharmacological methods. *J. Tradit. Complement. Med.* **11**, (2021).

17. Wang, Z. Z. *et al.* A small molecule compound berberine as an orally active therapeutic candidate against COVID-19 and SARS: A computational and mechanistic study. *FASEB J.* **35**, (2021).

18. Xie, T. A. *et al.* Identification of Hub genes associated with infection of three lung cell lines by SARS-CoV-2 with integrated bioinformatics analysis. *J. Cell. Mol. Med.* **24**, (2020).

19. Oh, J. H., Tannenbaum, A. & Deasy, J. O. Identification of biological correlates associated with respiratory failure in COVID-19. *BMC Med. Genomics* **13**, 1–7 (2020).

20. Vastrad, B., Vastrad, C. & Tengli, A. Identification of potential mRNA panels for severe acute respiratory syndrome coronavirus 2 (COVID-19) diagnosis and treatment using microarray dataset and bioinformatics methods. *3 Biotech* **10**, (2020).

21. Satu, M. S. *et al.* Diseasome and comorbidities complexities of SARS-CoV-2 infection with common malignant diseases. *Brief. Bioinform.* **00**, 1–15 (2021).

22. Taz, T. A. *et al.* Network-based identification genetic effect of SARS-CoV-2 infections to Idiopathic pulmonary fibrosis (IPF) patients. *Brief. Bioinform.* **22**, (2021).

23. Zhou, Y. *et al.* Network-based drug repurposing for novel coronavirus 2019-nCoV/SARS-CoV-2. *Cell Discov.* **6**, (2020).

24. Saxena, A. *et al.* A lung transcriptomic analysis for exploring host response in COVID-19. *J. Pure Appl. Microbiol.* **14**, 1077–1081 (2020).

25. Zhang, N., Zhao, Y. D. & Wang, X. M. CXCL10 an important chemokine associated with cytokine storm in COVID-19 infected patients. *Eur. Rev. Med. Pharmacol. Sci.* **24**, 7497–7505 (2020).

26. Wang, Z., Jiang, C., Zhang, X., Zhang, Y. & Ren, Y. Identication of Key Genes and Pathways in SARS-CoV-2 Infection using Bioinformatics Analysis. (2020) doi:10.21203/rs.3.rs-72821/v1.

27. Gu, H. & Yuan, G. Identification of potential key genes for SARS-CoV-2 infected human bronchial organoids based on bioinformatics analysis. *bioRxiv* (2020) doi:10.1101/2020.08.18.256735.

28. Soon Nan, K., Karuppanan, K., Kumar, S. & Alam, S. Identification of common key genes and pathways between Covid-19 and lung cancer by using protein-protein interaction network analysis. *bioRxiv* 2021.02.16.431364 (2021) doi:https://doi.org/10.1101/2021.02.16.431364.

29. Gu, H., Jiao, S. & Yuan, G. Identication of key genes and pathways in the hPSC-derived lungs infected by the SARS-CoV-2. (2020) doi:10.21203/rs.3.rs-114578/v1.

30. Sardar, R., Satish, D. & Gupta, D. Identification of Novel SARS-CoV-2 Drug Targets by Host MicroRNAs and Transcription Factors Co-regulatory Interaction Network Analysis. *Front. Genet.* **11**, 1–9 (2020).

31. Gu, H. & Yuan, G. Identification of key genes in SARS-CoV-2 patients on bioinformatics analysis. *bioRxiv* (2020) doi:10.1101/2020.08.09.243444.
